# Rapid biodegradation of atrazine by a novel *Paenarthrobacter ureafaciens* ZY and its effects on soil native microbial community dynamic

**DOI:** 10.3389/fmicb.2022.1103168

**Published:** 2023-01-04

**Authors:** Yue Zhao, Xin Li, Yunyang Li, Huanyu Bao, Jun Nan, Guoren Xu

**Affiliations:** ^1^School of Environment, Harbin Institute of Technology, Harbin, China; ^2^College of Resources and Environment, University of Chinese Academy of Sciences (UCAS), Beijing, China

**Keywords:** atrazine, *Paenarthrobacter ureafaciens*, degradation, bioremediation, native microbial communities

## Abstract

An atrazine-utilizing bacterium, designated as ZY, was isolated from agricultural soil and identified as *Paenarthrobacter ureafaciens*. The *P. ureafaciens* ZY demonstrated a significant degradation capacity of atrazine, with the degradation efficiency of 12.5 mg L^−1^ h^−1^ in liquid media (at pH 7, 30°C, and the atrazine level of 100 mg L^−1^). The *P. ureafaciens* ZY contained three atrazine-degrading genes (i.e., *trz*N, *atz*B, and *atz*C) could metabolize atrazine to form cyanuric acid, which showed lower biotoxicity than the parent atrazine as predicted by Ecological Structure Activity Relationships model. A laboratory-scale pot experiment was performed to examine the degradation of atrazine by *P. ureafaciens* ZY inoculation and investigate its effects on the native microbial communities. The results exhibited that the *P. ureafaciens* ZY was conductive to the degradation of atrazine, increased the total soil phospholipid fatty acids at the atrazine level of 50, 70, and 100 mg kg^−1^. By using high-throughput sequencing analysis, *Frateuria*, *Dyella*, *Burkholderia-Caballeronia-Paraburkholderia* were considered as the most important indigenous atrazine-degrading microorganisms due to their relative abundances were positively correlated with the atrazine degradation rate. In addition, *P. ureafaciens* ZY also increased the abundance of atrazine-degrading genus *Streptomyces* and *Bacillus*, indicating that there may be a synergic relationship between them in the process of atrazine degradation. Our work provides a new insight between inoculums and native microorganisms on the degradation of atrazine.

## 1. Introduction

The extensive use of pesticides significantly contributes to agricultural production by protecting crops from pests and diseases (Sun et al., 2018). According to the database from the Food and Agriculture Organization (FAO) of the United Nations (FAO, 2021), the annual consumption of pesticides reached 1.77 million tons (equal to 3.35 kg ha^−1^) in China in 2019, which far exceeded the maximum pesticides usage allowed levels with 1.66 kg ha^−1^. In fact, as little as 1% of applied pesticides reaches the target weeds and pests, and a more significant proportion of pesticides residues in the soils will lead to toxic effects on the ecological system and human health (Liang et al., 2022; Sabzevari and Hofman, 2022). In addition, pesticides that can spread in specific environments threaten the living organisms and result in an ecosystem imbalance (Lee and Choi, 2020; Lykogianni et al., 2021).

Atrazine (2-chloro-4-ethylamino-6-isopropylamino-1,3,5-triazine), a synthetic herbicide, has been extensively applied for more than 50 years. Its application has been controversial due to its widespread, high fluidity, and persistence, resulting in its high residues in soils (Sun et al., 2017). However, China and the United States still widely use atrazine, even though it has been restricted in the European Union and Canada (de Albuquerque et al., 2020). Moreover, the toxic effects of atrazine, such as neurotoxic, endocrine disrupting, and cancerogenic have been reported (Hayes et al., 2002; Shenoy, 2012; Shan et al., 2021). Many studies have been illustrated the toxic effects of atrazine on various organisms, e.g., *Caenorhabditis elegans* (García-Espiñeira et al., 2018; Zhou et al., 2021), *Chironomus tentans* (Tyler Mehler et al., 2008), *Daphnia carinata* (He et al., 2012), *Elodea canadensis* (Brain et al., 2012), *Lepomis macrochirus* (Tyler Mehler et al., 2008), *Litoditis marina* (Francolino et al., 2021), *Pimephales promelas* (Dionne et al., 2021), *Prochilodus lineatus* (de Andrade et al., 2019), and so on. Considering the long-term use of atrazine and its toxic effects, it is of great concern to explore a high-efficiency and safe strategy to restore the atrazine-contaminated environments.

Attribute to the efforts of mitigating the toxic effects of atrazine, a number of strains that can degrade atrazine have been isolated and characterized, so far, which seems to be good from a cost-effective, quality, degradation efficiency, and safety point of view. These atrazine-degrading strains isolated from different sources were reported, mainly including *Achromobacter* (Tonelli Fernandes et al., 2018), *Arthrobacter* (Bazhanov et al., 2017; Zhao et al., 2018), *Bacillus* (Jakinala et al., 2019), *Chelatobacter* (Rousseaux et al., 2003), *Citricoccus* (Yang et al., 2018), *Ensifer* (Chen et al., 2017), *Frankia* (Rehan et al., 2014), *Nocardia* (Giardi et al., 1985), *Nocardioides* (Omotayo et al., 2013). Besides the high degradation efficiency in the media (Rostami et al., 2021), atrazine-degrading strains were also inoculated into the atrazine-contaminated soil to explore their biodegradation ability of atrazine (Rousseaux et al., 2003; Sebaï et al., 2011; Gao et al., 2018). According to Li et al. (2008), 98.6%, 96.2%, and 88.7% of atrazine were removed by *Arthrobacter* sp. AD26, *A. aurescens* TC1, and *Pseudomonas* sp. ADP in atrazine-contaminated soil (300 mg kg^−1^) after inoculation for 20 days, respectively. A 96.86% degradation percentage of atrazine was observed with *Rhodobacter sphaeroides* sp. W16 inoculation in the soil at the level of 100 mg kg^−1^ atrazine after 15 days (Du et al., 2011). But up to now, most of the studies still focus on the isolation of new atrazine degrader and evaluation of atrazine degradation. However, a few researches have reported the effects of atrazine degraders inoculation on indigenous bacteria community in soil. Therefore, besides isolating safe and efficient atrazine-degrading strains, evaluation of the effects of atrazine-degrading strains on native microbial communities in atrazine-contaminated soil for the further application of these atrazine degraders was also essential.

In this study, a strain with a high-efficiency degradation capacity of atrazine, *P. ureafaciens* ZY, was isolated from agricultural soil in northeast China. The different liquid media parameters (i.e., pH, temperature, atrazine concentration) that affect atrazine degradation were explored. The genes and metabolic pathways involved in atrazine degradation by strain *P. ureafaciens* ZY were elucidated. Besides, this work also investigated the degradation capacity of *P. ureafaciens* ZY in the soil and its effects on the dynamic of soil native microbiome through PLFAs and high-throughput sequencing analysis. Our work enriched the species of bacteria that could degrade atrazine and provided a theoretical basis for the remediation of atrazine-contaminated soils.

## 2. Materials and methods

### 2.1. Soil samples, chemicals, and media

Soil samples were collected from agricultural soil in the northeast of China (126°49′40″ E, 45°40′60” N) and were sieved to remove plant residues and stones for further study (The main reason we selected this area was that it had long grown corn, which had also led to the widespread use of atrazine). Atrazine was purchased from Shanghai Macklin Biochemical Co., Ltd. Cyanuric acid was purchased from Heowns Biochem Technologies, LLC, Tianjin. The liquid minimal salt media (MSM) amended with glucose as a carbon source was consisted of 2.4 g K_2_HPO_4_, 0.2 g MgSO_4_·7H_2_O, 1.2 g KH_2_PO_4_, 0.025 g CaCl_2_·2H_2_O, 0.008 g Fe_2_ (SO_4_)_3_, and 1.0 g glucose in 1,000 ml of deionized water at pH 7.0 (marked as MSMG in this study). The liquid LB media contained 10 g tryptone, 5 g yeast extract, and 10 g NaCl in 1,000 ml of deionized water at pH 7.0. Solid media was formed by adding 17 g L^−1^ agar into liquid media, and all media were sterilized at 121°C for 30 min for further used.

### 2.2. Isolation of atrazine-degrading strains

Soil samples (10 g) were added to 100 ml of MSMG (contained 100 mg L^−1^ atrazine) and incubated under anaerobic conditions at 30°C and shaken at 160 rpm for 7 days. Then, the enrichment culture (10 ml) was transferred into a fresh MSMG (contained 200 mg L^−1^ atrazine) and incubated for 7 days at the same conditions. The inoculation process was repeated five times until the final atrazine concentration reached 500 mg L^−1^. Subsequently, the culture was collected and plated onto MSMG agar plates contained 100 mg L^−1^ of atrazine and incubated at 30°C in the darkness. After 4 days of incubation, single colonies were selected aseptically and explored their degradation ability for atrazine. A strain designated as ZY with atrazine degradation activity was obtained for further analysis.

### 2.3. Identification and characterization of strain ZY

The identified experiments of strain ZY, including morphological observation, 16S rRNA gene sequence analysis, and Biology Gene III MicroPlate, were performed. Bacteria Genomic DNA Kit (TransGene Biotech Co., Ltd., Beijing, China) was used to extract the total genomic DNA of strain ZY. Strain ZY was identified *via* 16S rRNA gene sequencing, the oligonucleotide primers and the PCR cycle parameters used as previously described by Yang et al. (2018). Then the 16S rRNA was sequenced by Shihe Biotech Co., Ltd. (Harbin, China). The 16S rRNA of strain ZY was compared to those 16S rRNA available in the National Center for Biotechnology Information (NCBI) *via* Nucleotide BLAST. MEGA version 8.0 was used to construct a phylogenetic tree. BIOLOG Gen III MicroPlate (BIOLOG Inc., Hay ward, USA) was carried out to test carbon substrates utilization patterns of strain ZY by using automated tetrazolium-based microbial identification system produced by BIOLOG Inc. (Hay ward, USA; Mekonnen et al., 2019).

### 2.4. Inoculum preparation

The strain ZY was cultured overnight (18 h) in LB media at 30°C and shaken at 160 rpm. After centrifugation, the strain cells were collected and suspended three times in MSMG media. A final cells density of 1 × 10^8^ CFU mL^−1^ (OD_600nm_ = 1.0) determined by a UV-spectrophotometer (Shanghai Jinghua Technology Instrument Co., Ltd) was obtained for subsequent degradation experiments.

### 2.5. Atrazine degradation of strain ZY in MSMG media

Atrazine degradation in liquid MSMG media was tested at pH 3.0, 5.0, 7.0, 9.0, and 11.0. The effect of temperature on atrazine degradation was tested at 20, 25, 30, 35, and 40°C. The degradation effects of the initial atrazine concentration were tested at 10, 30, 50, 70, and 100 mg L^−1^. Only one parameter was changed during each culture. The MSMG suspension with OD_600nm_ 1.0 were added into the fresh MSMG media at 1:50 (v:v) dosage of inoculation and cultured at 30°C. The OD_600nm_ and atrazine in the culture were detected every 2 h. Atrazine and cyanuric acid were determined by ultra-performance liquid chromatography (UPLC) analysis (Yang et al., 2018). The media without inoculation was used as controls, and the atrazine degradation was less than 1.0% in the controls.

### 2.6. Analysis of atrazine-degrading genes

The strain ZY was analyzed by PCR for the presence of atrazine-degrading genes (i.e., *atz*A, *atz*B, *atz*C, *atz*D, *atz*E, *atz*F, *trz*N, and *trz*D). The primer pairs used in this study were described in Supplementary Table S2, and the reaction conditions referred to Sebaï et al. (2011). Heshi Biotech Co., Ltd. (Harbin, China) performed the sequencing of atrazine-degrading genes, and the sequence analysis was done *via* BLAST on NCBI databases.

### 2.7. Determination and identification of atrazine metabolites

To identify the atrazine metabolites produced by *P. ureafaciens* ZY, the MSMG culture collected at 6 h was analyzed using a rapid and reliable ultra-high performance liquid chromatography-quadrupole-time-of-flight tandem mass spectrometry (UPLC-QTOF-MS, 6545 Q-TOF, Agilent Technologies, U.S.) technique. Specific UPLC-Q-TOF-MS experimental parameters and the mass spectrometry methods were performed as described by previous studies (Tonelli Fernandes et al., 2018; Zhao et al., 2018).

### 2.8. Biodegradation of atrazine in soil

The 200 g soil sample (dry weight) was added with methanol-dissolved atrazine to reach final concentrations of 10, 30, 50, 70, and100 mg kg^−1^ (dry weight) and then was placed in a fume hood for 4 h to volatilize methanol. The soils were inoculated with strain ZY to reach the initial concentration of 4 × 10^6^ CFU g^−1^, then passed through a mesh (2 mm) to achieve symmetrical. The treatments with ZY inoculums at 0, 10, 30, 50, 70, and 100 mg kg^−1^ atrazine were marked as P0, P10, P30, P50, P70 and P100, respectively. Soil samples containing atrazine at 0, 10, 30, 50, 70, and 100 mg kg^−1^ but without ZY inoculums were labeled as A0, A10, A30, A50, A70, A100, respectively. Then, soil samples were transferred to a plastic bowl (Φ 9 cm) and incubated in darkness at 25°C with 30% water-holding content adjusted by sterile distilled water. After inoculation for 1, 3, 5, and 7 d, soil samples were collected to analyze residual atrazine. In brief, 3 g of soil samples were added with 15 ml methanol for ultrasonic for 20 min, followed by centrifugation for supernatant collection, and the above operations were repeated three times (Luo et al., 2021). The received supernatant was concentrated by a rotary evaporator and dissolved by chromatographic methanol, followed by 0.22 μm filter membrane filtration for UPLC analysis.

### 2.9. The PLFAs analysis

The PLFAs analysis was used to assess soil microbial community structures. The PLFAs were extracted and analyzed using the methods reported by Frostegård et al. (1993) and Chaudhary et al. (2020). The Sherlock Microbial Identification System (SMIS), developed by MIDI Corporation (MIDI, Newark, Delaware, USA), was used to identify PLFAs in soil. All PLFAs classifications were shown in Supplementary Table S4 provided by MSIS.

### 2.10. Soil DNA extraction, sequencing and bioinformatics analysis

The Illumina MiSeq of PCR-amplified 16S rRNA was used to analysis the structure of soil microbial community. Total DNA of soil was extracted by using the Cetyltrimethyl Ammonium Bromide (CTAB) according to manufacturer’s instructions. The 16S rRNA gene fragment was amplified using a universal primer set 341F (5’-CCTACGGGNGGCWGCAG-3′) and 805R (5’-GACTACHVGGGTATCTAATCC-3′; Logue et al., 2016). Sequencing was performed by LC-Bio Technology Co., Ltd., Hang Zhou, Zhejiang Province, China. The raw reads have been submitted to NCBI with the SRA database accession of PRJNA909019.

All experiments were performed in triplicate, and the results were presented as arithmetic averages and standard deviations. The datas were analyzed with the SPSS 19.0 and Origin 18.0 software. The Ecological Structure Activity Relationships (ECOSAR) model was used to predict the toxicity of atrazine metabolites. Principal component analysis (PCA) was performed by CANOCO 5.0 software. Heat map analysis and network analysis were performed using the R package (v3.5.2).

## 3. Results and discussion

### 3.1. Isolation and characterization of strain ZY

A strain ZY with a strong atrazine-degrading ability was selected for further study. A light yellow, smooth, and round colony of strain ZY was observed in the LB plate (Supplementary Figure S1A). Its morphological characteristics indicated that strain ZY was a Gram positive, and rod-shaped bacteria (Supplementary Figures S1B, S2).

**Figure 1:**
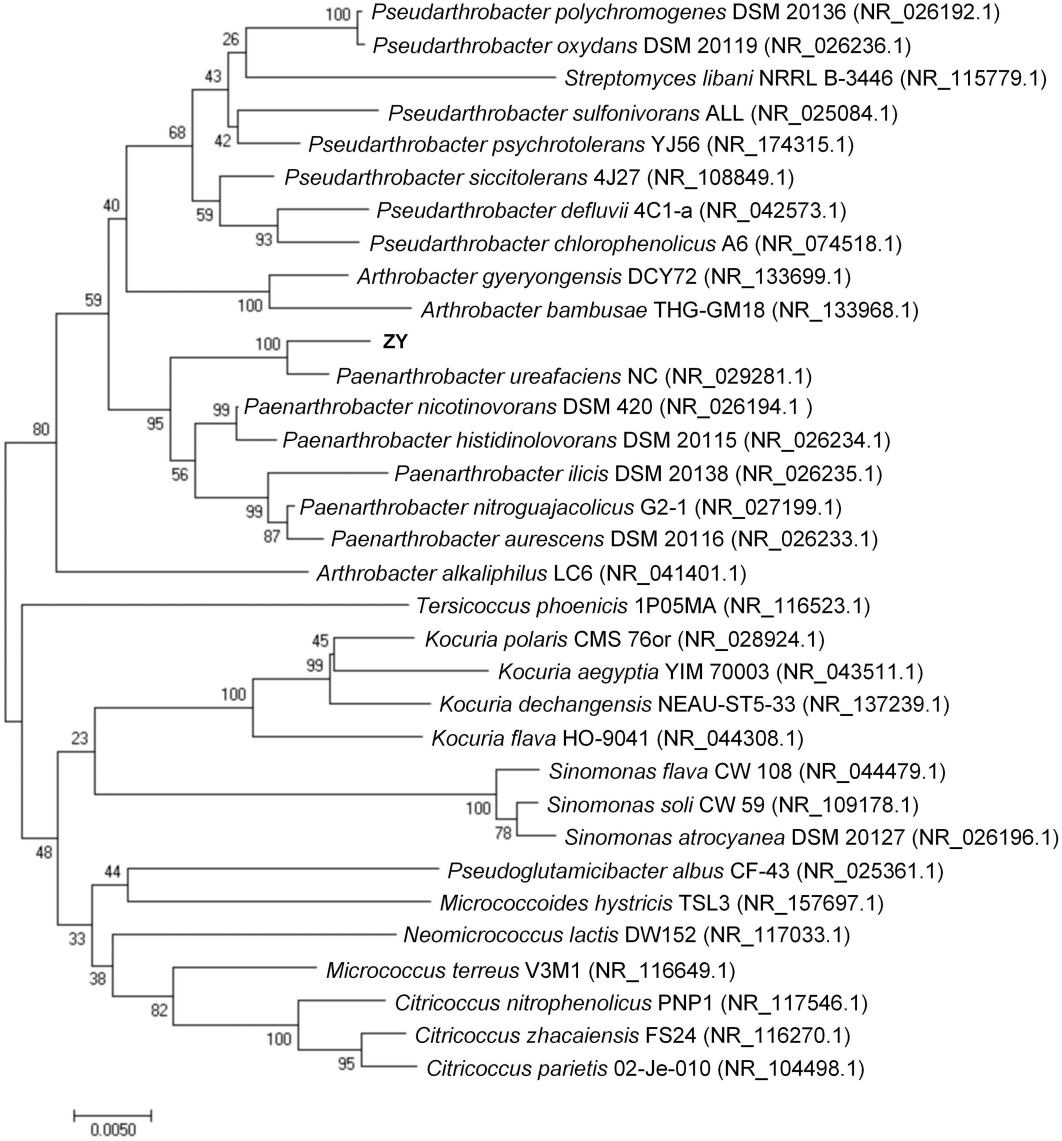
Phylogenetic tree based on the 16S rRNA gene sequences of strain ZY. The calculations were performed according to a neighbor-joining analysis (bootstrap number = 1,000), and the bar indicates 0.0050 substitution per nucleotide position.

The 16S rRNA gene sequence of strain ZY was 1,376 bp (accession no. ON878081), very similar to those of accessible strains, e.g., *P. ureafaciens* NC (99.19%, accession no. NR_029281.1), *P. nicotinovorans* DSM 420 (98.37%, accession no. NR_026194.1), and *P. histidinolovorans* DSM 20115 (98.21%, accession no. NR_026234.1). In a phylogenetic tree constructed based on the similar 16S rRNA gene sequences of ZY (Figure 1), the strain ZY clustered with members of the genus *Paenarthrobacter* sp. and was preliminarily identified as a *P. ureafaciens* due to the closest relative with *P. ureafaciens* NC.

Carbon utilization pattern of *P. ureafaciens* ZY was investigated *via* GEN III microplate after 48 h of incubation. The results have shown in Supplementary Table S3, Figure S3. Accordingly, *P. ureafaciens* ZY can metabolize D-fructose, D-mannose, D-galactose as carbon sources, but cannot assimilate dserine, troleandomycin, rifamycin, and minocycline (Supplementary Table S3).

### 3.2. The effects of culture conditions on the growth and atrazine degradation of *Paenarthrobacter ureafaciens* ZY

In the liquid MSMG, the effects of pH, temperature, and atrazine concentration on the growth and atrazine degradation of *P. ureafaciens* ZY were investigated.

#### 3.2.1. pH

**Figure 2:**
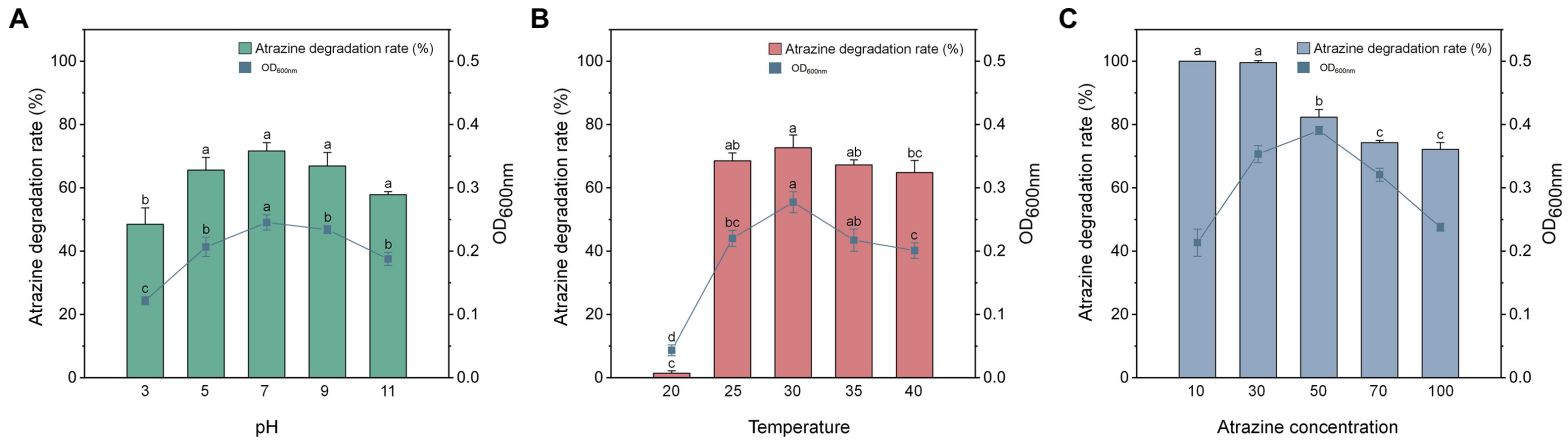
The effects of pH **(A)**, temperature **(B)**, and atrazine concentration **(C)** on the atrazine degradation and growth of *Paenarthrobacter ureafaciens* ZY.

Environmental pH could influence the microbial remediation of contaminated sites (Siddique et al., 2002). The optimal pH range for the growth and atrazine degradation by *Arthrobacter* sp. strain HB-5 was 5.0–10.0 (Wang and Xie, 2012). Figure 2A displayed the growth and degradation of atrazine of *P. ureafaciens* ZY in MSMG (100 mg L^−1^ atrazine) at different pH levels for 6 h. After inoculation for 6 h, more than 64.83% of atrazine removal were observed at pH 5.0–11.0. At pH 3.0, *P. ureafaciens* ZY hardly grew and could not metabolize atrazine. The degradation rates of atrazine were 11.42, 12.11, 11.21, and 10.81 mg L^−1^ h^−1^ at pH ranging from 5 to 11, respectively. The pH range for the growth and atrazine degradation of *P. ureafaciens* ZY is wide and alkaliphilic, which also revealed that *P. ureafaciens* ZY had a great potential to rete atrazine-contaminated sites, particularly in alkaline sites. In addition, there was a significant correlation between the atrazine degradation percentage and OD_600nm_ from pH 3 to 11 (*r* = 0.953, *p* < 0.01), suggesting that pH was an important factor affecting strain proliferation and atrazine degradation.

#### 3.2.2. Temperature

Temperature has played a key role in biodegradation efficiency of pollutants by microorganisms, and significantly influences the dissipation of s-triazine herbicides (Vela et al., 2004). Figure 2B showed the effects of temperature ranging from 20°C to 40°C on the degradation of 100 mg L^−1^ atrazine in MSMG (pH 7.0). With the increase of temperature, the degradation of atrazine enhanced and degradation rates were 8.08, 8.20, and 11.95 mg L^−1^ h^−1^ at 20, 25, and 30°C after 6 h, respectively. Notably, the degradation ability of atrazine were inhibited at 35°C and 40°C after 6 h. Yang et al. (2018) also found that the optimum temperature for the growth and degradation of *Citricoccus* sp. strain TT3 was 30°C, and the growth and atrazine degradation of *Citricoccus* sp. strain TT3 were inhibited at 37°C and 45°C. In addition, a significant correlation between the atrazine degradation percentage and OD_600nm_ from 20 to 40°C (*r* = 0.869, *p* < 0.01) was observed, indicating that strain proliferation and atrazine degradation could be affected by the temperature.

#### 3.2.3. Atrazine concentrations

The effects of atrazine concentrations at the levels of 10–100 mg L^−1^ on the growth and atrazine degradation of *P. ureafaciens* ZY were explored in MSMG at pH 7.0 and 30°C (Figure 2C). After 6 h inoculation of *P. ureafaciens* ZY, the degradation rates of atrazine were approximately 100, 99.57, 82.33, 74.28, and 72.17% at the atrazine levels of 10, 30, 50, 70, 100 mg L^−1^, respectively (Figure 2C). After the inoculation of 8 h, atrazine was completely degraded in all groups. According to OD_600nm_ of *P. ureafaciens* ZY, with the increase of atrazine concentration (from 10 to 50 mg L^−1^), the growth of *P. ureafaciens* ZY was obviously promoted (Figure 2C). The concentrations of atrazine ranging from 70 to 100 mg L^−1^ inhibited the growth and atrazine degradation of *P. ureafaciens* ZY, which might due to the toxicity of high concentrations of atrazine or their metabolites to *P. ureafaciens* ZY (Zhao et al., 2018).

The degradation rate of *P. ureafaciens* ZY in 8 h at an atrazine concentration of 100 mg L^−1^ was 12.5 mg L^−1^ h^−1^, which was 27.78, 10.94, and 4.50 times greater than those of *Rhodococcus* sp. BCH2, *Pseudomonas* sp. EGD-AKN5, and *Shewanella* sp. YJY4, respectively (Supplementary Table S4). Even in close relatives *Arthrobacter*, *P. ureafaciens* ZY exhibited an increase in the degradation rate of 10.42 mg L^−1^ h^−1^ and 2.98 mg L^−1^ h^−1^ than those in *Arthrobacter* sp. DAT1 and *Arthrobacter* sp. ZXY-2 at the atrazine level of 100 mg L^−1^. Compared to the model atrazine-degrading bacterium *Pseudomonas* sp. ADP (Mandelbaum et al., 1995), *P. ureafaciens* ZY still shows an approximately 8.33 mg L^−1^ h^−1^ increase of degradation rate with 100 mg L^−1^ atrazine. To our best knowledge, *P. ureafaciens* ZY showed the highest degradation capacity of atrazine compared to the other reported atrazine-degrading strains at the atrazine level ranges from 50 to 100 mg L^−1^. These results illustrated that *P. ureafaciens* ZY had a great bioretion potential, especially for the heavily atrazine-contaminated sites.

### 3.3. Atrazine-degrading genes and the metabolites of atrazine

At present, atrazine degradation genes have been extensively investigated and proven to be highly conserved in many bacteria (de Souza et al., 1998; Singh and Jauhari, 2017). According to the different genetic compositions, atrazine-degrading bacteria are divided into two types. One type only contains *atz*A (*trz*N), *atz*B, and *atz*C, which could catalyze atrazine to produce cyanuric acid (Zhao et al., 2018). And the other type used extra genes *atz*D (*trz*D), *atz*E, and *atz*F to mineralize atrazine into CO_2_ and NH_3_ (Ma et al., 2017).

**Figure 3:**
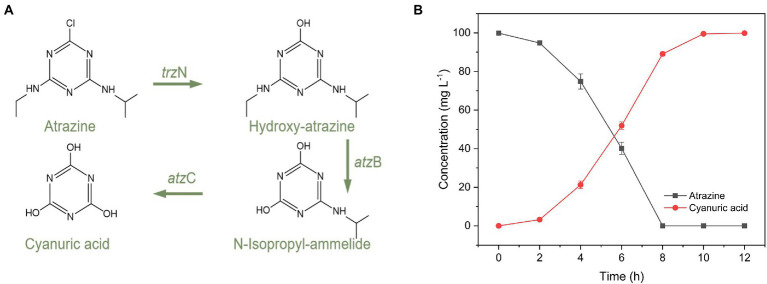
Concentration changes in atrazine and cyanuric acid **(A)**. The proposed metabolic pathway of atrazine in *P. ureafaciens* ZY **(B)**.

In this work, according to the primers in Supplementary Table S1, three genes including *trz*N, *atz*B, and *atz*C were obtained in *P. ureafaciens* ZY (Supplementary Figure S6). The amplified gene sequences were compared with those in NCBI database. The *trz*N (438 bp, ON954325) exhibited 100% identity with the *trz*N gene of *Arthrobacter* sp. AK-YN10 (HE716868.1) and *Pseudomonas* sp. ADP (FJ161692.1). The *atz*B (1,391 bp, ON911740) showed 100% homologous with the *atz*B gene of *Arthrobacter* sp. ZXY-2 (CP017421.1) and *P. ureafaciens* DnL1-1 (CP014574.1). The *atz*C (616 bp, ON954326) showed 99.67 and 99.67% homologous with the *atz*C gene of *Pseudomonas* sp. ADP (U66917.2) and *Nocardioides* sp. SP12 (AF537329.1). During the degradation process of atrazine by *P. ureafaciens* ZY, three metabolites of atrazine, were identified as hydroxyatrazine (m/z of 198.0), N-isopropylammelide (m/z of 171.0), and cyanuric acid (m/z of 128.0) by UPLC-Q-TOF-MS (Supplementary Figure S4). In Figure 3A, a putative atrazine degradation pathway was proposed: In the first step, hydrolytic dechlorination was induced by triazine hydrolase which endoded by *trz*N to yield hydroxyatrazine. Subsequently, hydroxyatrazine ethylaminohydrolase which encoded by *atz*B catalyzes hydroxyatrazine to form N-isopropylammelide. Finally, the N-isopropylammelide was eliminated from the s-triazine ring which induced by N-isopropylammelide isopropylamidohydrolase (encoded by *atz*C) to form cyanuric acid. Further determination of cyanuric acid content can also confirm the degradation path of atrazine (Figure 3B). The atrazine degradation pathway of *P. ureafaciens* ZY is similar to that in *Arthrobacter* sp. ZXY-2 (Zhao et al., 2018) and *Citricoccus* sp. strain TT3 (Yang et al., 2018), and different from other atrazine degraders which can mineralize atrazine, such as *Ensifer* sp. CX-T (Ma et al., 2017) and *Pseudomonas* sp. ADP (Martinez et al., 2001). By using the ECOSAR model, we also predicted the biotoxicity of the metabolites of atrazine, and the results were shown in Supplementary Table S5. Hydroxyatrazine, N-isopropylammelide, and cyanuric acid showed lower biotoxicity than its parent atrazine. Kolekar et al. (2019) also evaluated cytotoxicity tests by cell viability assay on HepG2 and indicated significant decrease in the toxicity of atrazine by the atrazine-degrading strains inoculation due to its effective degradation and formation of simpler and less/nontoxic metabolites (Hydroxyatrazine and N-isopropyllamilide). Intracellular reactive oxygen species levels and malondialdehyde content exhibited a significantly decreasing tendency after *Escherichia coli* exposure to intermediates (Hydroxyatrazine, N-isopropyllamilide, and cyanuric acid) of atrazine compared to parent atrazine (Yu et al., 2023). In conjunction, these results indicated that *P. ureafaciens* ZY had practical application value.

### 3.4. The removal of atrazine in soil with *Paenarthrobacter ureafaciens* ZY inoculation

**Figure 4:**
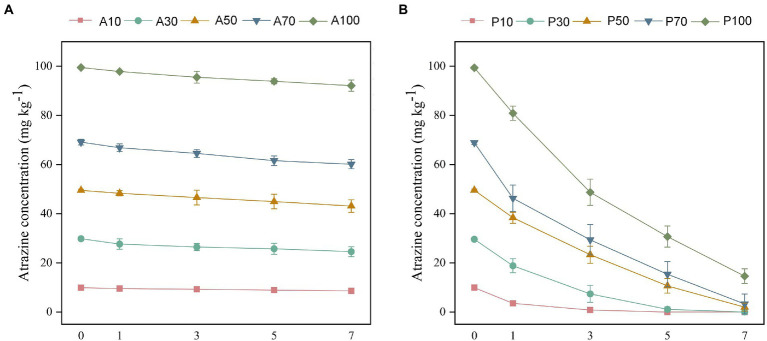
The concentrations of atrazine residues without *P. ureafaciens* ZY inoculation **(A)** and with *P. ureafaciens* ZY inoculation **(B)** treatments after 7 days. A10: 10 mg kg^−1^ atrazine-contaminated soil; A30: 30 mg kg^−1^ atrazine-contaminated soil; A50: 50 mg kg^−1^ atrazine-contaminated soil; A70: 70 mg kg^−1^ atrazine-contaminated soil; A100: 100 mg kg^−1^ atrazine-contaminated soil; P10: 10 mg kg^−1^ atrazine-contaminated soil with *P. ureafaciens* ZY inoculation; P30: 30 mg kg^−1^ atrazine-contaminated soil with *P. ureafaciens* ZY inoculation; P50: 50 mg kg^−1^ atrazine-contaminated soil with *P. ureafaciens* ZY inoculation; P70: 70 mg kg^−1^ atrazine-contaminated soil with *P. ureafaciens* ZY inoculation; P100: 100 mg kg^−1^ atrazine-contaminated soil with *P. ureafaciens* ZY inoculation.

The biodegradation of different concentrations of atrazine was further investigated in soil with or without *P. ureafaciens* ZY inoculation. Without *P. ureafaciens* ZY inoculation, the highest degradation of atrazine (17.66%) was found in A30 after 7 days. In the treatments of A10, A50, A70, and A100, the degradation of atrazine was 12.71%, 12.87%, 13.03%, and 7.44%, respectively (Figure 4A). Atrazine was not detected in A0 and P0. Complete degradation of atrazine at 10 and 30 mg kg^−1^ occurred within 5 days and 7 days in soil inoculated with *P. ureafaciens* ZY. In P50, P70, and P100, the degradation of atrazine at 7 days was 95.96%, 95.21%, and 85.31%, respectively (Figure 4B). In recent years, *Paenarthrobacter* has been screened and demonstrated strong atrazine-degrading ability. Jia et al. (2021) found that 95.9% of 5 mg kg^−1^ atrazine was removed from the soils when inoculated with *Paenarthrobacter* strain AT-5 with 7 days. Chen et al. (2021) also reported that 96.0% of atrazine had been degraded with *Paenarthrobacter* sp. W11 inoculation after 49 days of incubation in soils (50 mg kg^−1^ atrazine). These results indicated that *P. ureafaciens* had great potential for remediation of atrazine-contaminated soil.

In this work, the freshly added soils were used, and the addition of *P. ureafaciens* ZY showed a high degradation rate for atrazine in soil. Zhao et al. (2003) revealed that the degradation percentage of atrazine in the freshly added soils was significantly higher than that in the aged soils. The degradation efficiency decreased with the extension of inoculation time, and the half-life of atrazine inoculated in aged soil was longer than that in the freshly added soil. This can be explained by the fact that the bioavailability of atrazine in the freshly added soils is obviously increased compared to that in the aged soils (Yanze-Kontchou and Gschwind, 1995; Radosevich et al., 1997; Zhao et al., 2003).

### 3.5. The soil PLFAs dynamic following with *Paenarthrobacter ureafaciens* ZY inoculation

**Figure 5:**
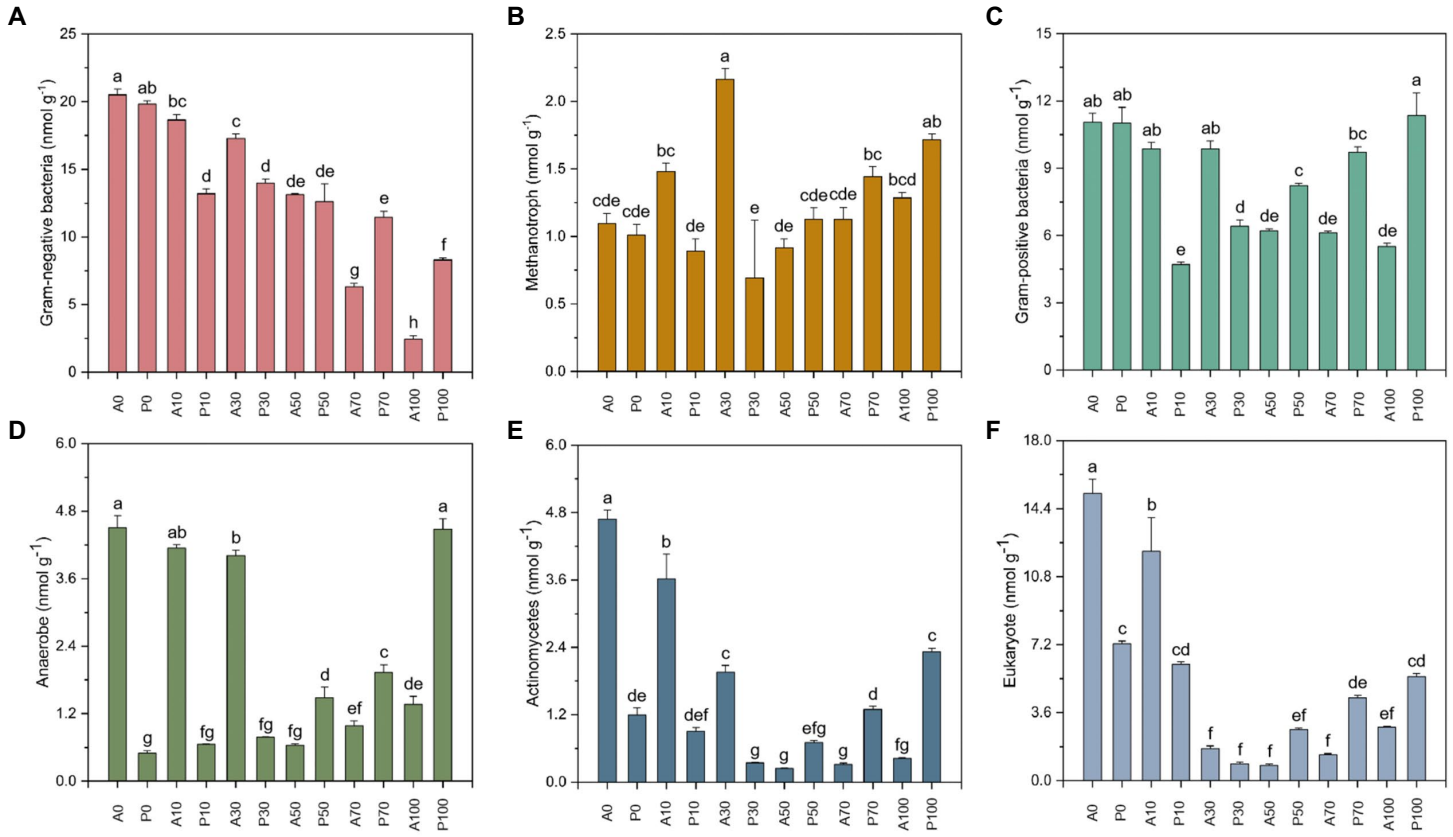
The PLFAs (nmol g^−1^) indicated by Gram-negative **(A)**, Methanotroph **(B)**, Gram-positive **(C)**, Anaerobe **(D)**, Actinobacteria **(E)**, and Eukaryote **(F)** in different treatments on day 7. A0: Equivalent amount of methanol were added into native soil. A10: 10 mg kg^−1^ atrazine-contaminated soil; A30: 30 mg kg^−1^ atrazine-contaminated soil; A50: 50 mg kg^−1^ atrazine-contaminated soil; A70: 70 mg kg^−1^ atrazine-contaminated soil; A100: 100 mg kg^−1^ atrazine-contaminated soil; P0: Equivalent amount of methanol and *P. ureafaciens* ZY were added into native soil; P10: 10 mg kg^−1^ atrazine-contaminated soil with *P. ureafaciens* ZY inoculation; P30: 30 mg kg^−1^ atrazine-contaminated soil with *P. ureafaciens* ZY inoculation; P50: 50 mg kg^−1^ atrazine-contaminated soil with *P. ureafaciens* ZY inoculation; P70: 70 mg kg^−1^ atrazine-contaminated soil with *P. ureafaciens* ZY inoculation; P100: 100 mg kg^−1^ atrazine-contaminated soil with *P. ureafaciens* ZY inoculation.

Figure 5 exhibited the abundance of PLFAs (i.e., the biomass of microorganisms) in different treatments. In non-inoculation treatments, the total PLFAs of A0 (123.026 nmol g^−1^) is higher than those in other treatments, especially in A70 (37.948 nmol g^−1^, *p* < 0.01) and A100 (14.579 nmol g^−1^, *p <* 0.01). Among them, the Gram-positive (Figure 5A), Gram-nagative (Figure 5B), Methanotroph (Figure 5C) and Actinomycetes (Figure 5E) also showed the same decrease trend. Previous studies have demonstrated that a specific inhibitory effect on soil microbes occurred by atrazine addition (Ros et al., 2006; Gao et al., 2018; Chen et al., 2021). Gao et al. (2018) also indicated that the utilization of carbon sources by microbial communities was decreased in atrazine-contaminated soil. The decline of soil microbial biomass might be caused by the accumulated metabolites, which might be more toxic to the microbes than the parent in the soil (Cheng et al., 2022). Seghers et al. (2003) revealed that atrazine significantly affected the structure and function of soil microbial community. Besides, the inhibition effects on the soil community and nitrification process were also reported (Chen et al., 2015).

At relatively low concentrations of 10–30 mg kg^−1^ of atrazine, the total PLFAs of the non-inoculated groups were higher than that in the inoculated groups, the decrease of PLFAs of 32.687 nmol g^−1^ and 19.858 nmol g^−1^ were found in A10 and A30 compared to P10 and P30. And the same decrease trend was observed in Gram-negative (Figure 5A), Methanotroph (Figure 5B), Gram-positive (Figure 5C), Anaerobe (Figure 5D), Actinomycetes (Figure 5E) and Eukaryote (Figure 5F), which might be caused by competition between *P. ureafaciens* ZY and indigenous microorganisms (Abtahi et al., 2020; Koolivand et al., 2022). On the contrary, the biostimulation of *P. ureafaciens* ZY led to a significant increase of PLFAs at atrazine concentration of 50–100 mg kg^−1^, especially for Gram-negative (Figure 5A), Gram-positive (Figure 5C), Anaerobe (Figure 5D), Actinomycetes (Figure 5E) in 100 mg kg^−1^ atrazine treatments. This result was consistent with Gao et al. (2018) and Chen et al. (2021), which was possibly related with two reasons First, with the continuous removal of atrazine after inoculation of *P. ureafaciens* ZY, the inhibition effect of atrazine on soil microorganisms gradually decreased (Ros et al., 2006). Secondly, most microorganisms in the soil could not have the abllity to metabolize atrazine and cannot directly absorb atrazine as a carbon source and nitrogen source (Singh and Jauhari, 2017). After the decomposition of atrazine by *P. ureafaciens* ZY, atrazine metabolites could be acted as nitrogen and carbon source by soil microorganisms to meet their growth and proliferation (Chen et al., 2021).

### 3.6. Effect of *Paenarthrobacter ureafaciens* ZY on soil bacterial community dynamics

**Figure 6:**
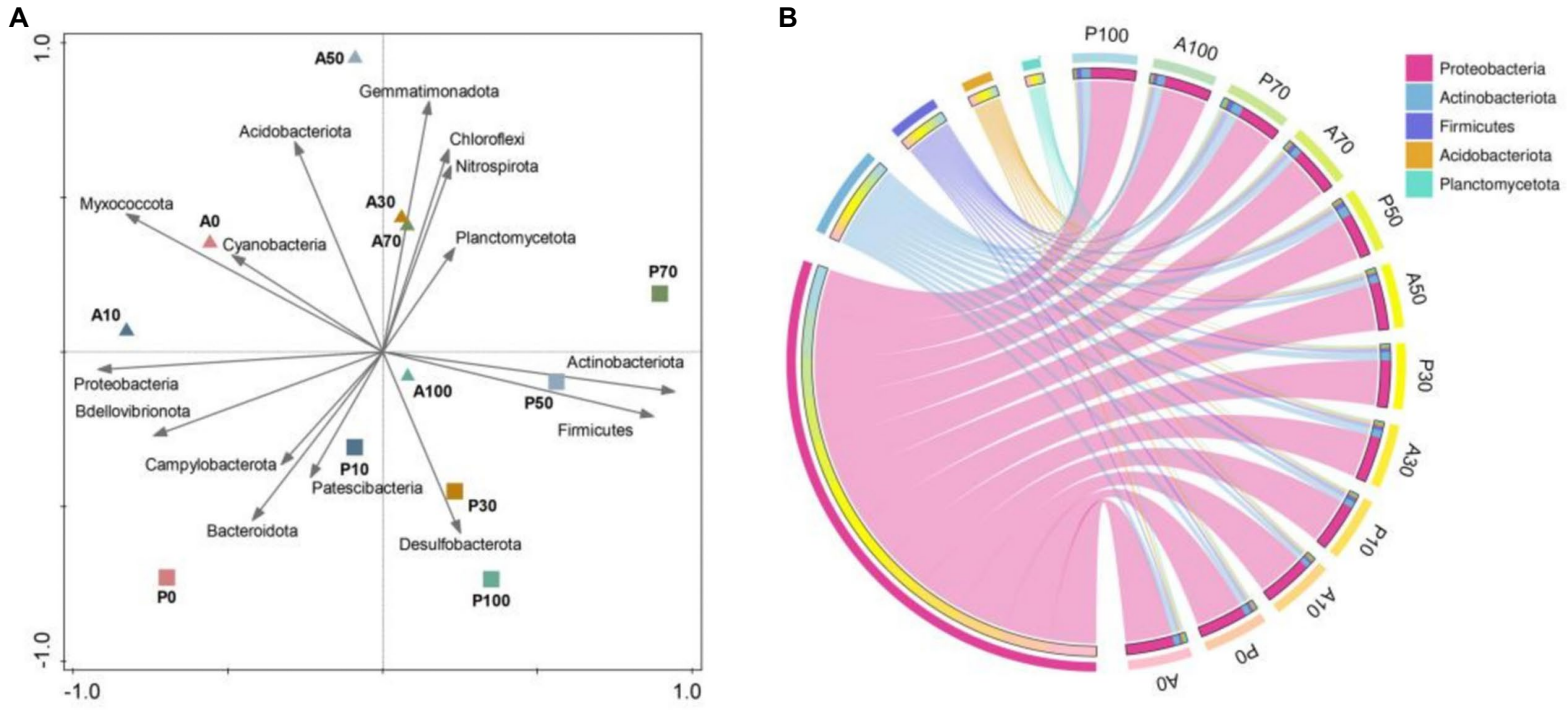
Effects of the different treatments on the soil bacterial community structure. Principal component analysis (PCA) based on the top 15 phylum of each sample **(A)**; Chord diagram based on relative abundances of bacterial phylum in different treatments **(B)**. A0: Equivalent amount of methanol were added into native soil. A10: 10 mg kg^−1^ atrazine-contaminated soil; A30: 30 mg kg^−1^ atrazine-contaminated soil; A50: 50 mg kg^−1^ atrazine-contaminated soil; A70: 70 mg kg^−1^ atrazine-contaminated soil; A100: 100 mg kg^−1^ atrazine-contaminated soil; P0: Equivalent amount of methanol and *P. ureafaciens* ZY were added into native soil; P10: 10 mg kg^−1^ atrazine-contaminated soil with *P. ureafaciens* ZY inoculation; P30: 30 mg kg^−1^ atrazine-contaminated soil with *P. ureafaciens* ZY inoculation; P50: 50 mg kg^−1^ atrazine-contaminated soil with *P. ureafaciens* ZY inoculation; P70: 70 mg kg^−1^ atrazine-contaminated soil with *P. ureafaciens* ZY inoculation; P100: 100 mg kg^−1^ atrazine-contaminated soil with *P. ureafaciens* ZY inoculation.

The high-throughput sequencing was applied to investigate native microbial community dynamics at 7 days in different treatments. The OTU (Supplementary Figure S5A), Chao1 (Supplementary Figure S5B), Simpson (Supplementary Figure S5C) and Shannon (Supplementary Figure S5D) indexes of the bacterial community in A0 treatment were higher than that in other treatments at 7 days. These results indicated that atrazine and inoculation of *P. ureafaciens* ZY reduced the soil community richness and diversity. Jia et al. (2021) reported that the bioaugmentation of atrazine-contaminated soil with strain AT5 significantly decreased the bacterial richness and diversity, which might be due to the persistence and niche occupation of inoculation strain. In addition, *P. ureafaciens* ZY inoculation was also associated with interte production and nutrient consumption during the metabolism of atrazine, leading to the changes of soil micro-environment. Yang et al. (2021) have shown that the changes in the micro-environment could result in a significant decrease in bacterial richness and diversity. PCA analysis (Figure 6A) showed that both the bacterial community structure and the relative abundance of bacteria at the phylum level in A0 is similar to those in atrazine alone addition, and different from those in *P. ureafaciens* ZY inoculation treatments. It indicated that inoculums rather than atrazine was the main factor affecting the bacterial community structure (Jia et al., 2021).

In our study, Proteobacteria, Actinobacteriota, Firmicutes, Acidobacteriota, Planctomycetota were the primary bacterial phyla in the soil samples (Figure 6B). Previous study found that Proteobacteria are the dominant microorganisms in atrazine-contaminated soils (Jia et al., 2021). The members of phylum, including Actinobacteria, Firmicutes, and Proteobacteria were reported to be the best sources for degradation of pesticides (Kumar et al., 2021). The reported atrazine degrading bacteria were mainly distributed in Actinobacteria, Firmicutes, and Proteobacteria, such as, *Achromobacter* (Proteobacteria)*, Arthrobacter/Citricoccus* (Actinobacteria), *Bacillus* (Firmicutes), respectively (Tonelli Fernandes et al., 2018; Yang et al., 2018; Zhao et al., 2018; Jakinala et al., 2019).

**Figure 7:**
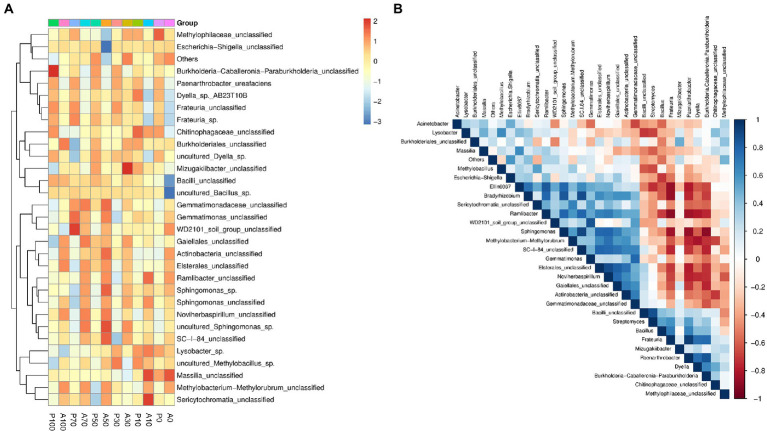
Heatmaps showing relative abundances of abundant genera **(A)**; Spearman rank correlation obtained by the analysis of top 30 genera in different treatments at 7 days **(B)**; Network A0: Equivalent amount of methanol were added into native soil. A10: 10 mg kg^−1^ atrazine-contaminated soil; A30: 30 mg kg^−1^ atrazine-contaminated soil; A50: 50 mg kg^−1^ atrazine-contaminated soil; A70: 70 mg kg^−1^ atrazine-contaminated soil; A100: 100 mg kg^−1^ atrazine-contaminated soil; P0: Equivalent amount of methanol and *P. ureafaciens* ZY were added into native soil; P10: 10 mg kg^−1^ atrazine-contaminated soil with *P. ureafaciens* ZY inoculation; P30: 30 mg kg^−1^ atrazine-contaminated soil with *P. ureafaciens* ZY inoculation; P50: 50 mg kg^−1^ atrazine-contaminated soil with *P. ureafaciens* ZY inoculation; P70: 70 mg kg^−1^ atrazine-contaminated soil with *P. ureafaciens* ZY inoculation; P100: 100 mg kg^−1^ atrazine-contaminated soil with *P. ureafaciens* ZY inoculation.

Figure 7A shows the shift in the relative abundances of bacterial genera. The inoculated genus *Paenarthrobacter* was not been detected in the atrazine alone treatments due to its low abundance. The relative abundances of *Paenarthrobacter* were 1.08% (P0), 1.11% (P10), 2.56% (P30), 2.75% (P50), 4.39% (P70) and 3.30% (P100) at 7 days, respectively. These results showed that the inoculated *P. ureafaciens* ZY could survive and proliferate in atrazine-contaminated soils. In our study, the atrazine degradation rate was positively correlated with *Frateuria* (*p <* 0.01), *Dyella* (*p <* 0.05), *Burkholderia-Caballeronia-Paraburkholderia* (*p <* 0.05), and *Paenarthrobacter* (*p <* 0.01), respectively. Compared to A0, an increase of relative abundance of 16.71%–312.53% (*Frateuria*) and 7.09%–66.93% (*Dyella*) were found in other groups. Atrazine alone addition decreased the relative abundance by 1.49%–26.73% in *Burkholderia-Caballeronia-Paraburkholderia*, however, *P. ureafaciens* ZY increased the relative abundance of *Burkholderia-Caballeronia-Paraburkholderia* by 31.68%–221.78% compared to those in non-inoculation treatments. The previous studies had reported that *Frateuria* species could metabolize amine (Aoki et al., 1984) and *Paraburkholderia lycopersici* sp. nov., was involved in soil nitrogen metabolism (Tapia-García et al., 2020). *Dyella* also showed excellent ability to degrade pollutants (Li et al., 2009; Cai et al., 2022). In conclusion, *Dyella*, *Frateuria* and *Burkholderia-Caballeronia-Paraburkholderia* may be important genera involved in the metabolism of atrazine and its metabolites. In addition, as shown in Figure 7B*. Paenarthrobacter ureafaciens* ZY increased the abundance genus *Streptomyces* and *Bacillus*, and there is a positive correlation between *P. ureafaciens* ZY, *Streptomyces* and *Bacillus.* In the previous study, *Bacillus* showed atrazine degradation ability (Jakinala et al., 2019) and the inoculation of biomixtures with *Streptomyces* sp. M7 increased atrazine removal (Saez et al., 2022). These results suggested that there might be a synergistic relationship between *P. ureafaciens* ZY, *Bacillus* and *Streptomyces* during atrazine degradation.

In addition, to further characterize the effects of *P. ureafaciens* ZY inoculation on soil native microbial community. The co-occurrence patterns of bacterial communities were assessed (Supplementary Figure S7). The more complex and well-connected co-occurrence networks were observed in atrazine alone treatments indicated by edge numbers and density with the value of 500 and 0.025 (Supplementary Table S8). This occurrence might be due to the stimulation of atrazine on the soil microbial diversity. The bacterial taxonomic composition of “hub nodes” in the network differed between atrazine alone treatments and *P. ureafaciens* ZY inoculation treatments. OTUs belonging to *Sphingomonas* in *P. ureafaciens* ZY inoculation treatments, and OTUs belonging to *Gaiellales* and *Sericytochromatia* in atrazine alone treatments were more connected with others (Supplementary Figure S7). Guo et al. (2022) found that hub species in microbial networks are recognized as mediators among associated microbiome. Trough network hubs, it follows that *P. ureafaciens* ZY inoculation may influence the assembly of soil native microbial community by regulating microbe-microbe interactions (Trivedi et al., 2020).

## 4. Conclusion

This study isolated and identified a novel atrazine degrader *P. ureafaciens* ZY. *Paenarthrobacter ureafaciens* ZY exhibited a significant degradation capacity of atrazine both in liquid media and soil. The *P. ureafaciens* ZY could metabolize atrazine to form cyanuric acid *via* related enzymes which encoded by *trzN*, *atz*B, and *atz*C. Besides, *P. ureafaciens* ZY increased the biomass of native bacterial communities and restored the microbial abundance and diversity in heavily atrazine-contaminated soil (50–100 mg kg^−1^ atrazine). In addition, *Frateuria*, *Dyella*, and *Burkholderia-Caballeronia-Paraburkholderia* showed significantly positive correlations with atrazine degradation rate, suggesting that they were also important participants in the atrazine degrading process. Moreover, there may be a synergic relationship between *P. ureafaciens* ZY, *Streptomyces* and *Bacillus* during atrazine degradation. Our work provides a theoretical basis for bioaugmentation in atrazine-contaminated soil.

## Data availability statement

The datasets presented in this study can be found in online repositories. The names of the repository/repositories and accession number (s) can be found in the article/Supplementary material.

## Author contributions

YZ: conceptualization, investigation, data curation, Writing—original draft. XL: supervision, funding acquisition, writing—review. YL: methodology, writing. HB: methodology, writing. JN: supervision, Writing—review and editing. GX: Writing—review and editing. All authors contributed to the article and approved the submitted version.

## Funding

National Key Research and Development Program of China (2019YFC1906501 and 2018YFC1901100).

## Conflict of interest

The authors declare that the research was conducted in the absence of any commercial or financial relationships that could be construed as a potential conflict of interest.

## Publisher’s note

All claims expressed in this article are solely those of the authors and do not necessarily represent those of their affiliated organizations, or those of the publisher, the editors and the reviewers. Any product that may be evaluated in this article, or claim that may be made by its manufacturer, is not guaranteed or endorsed by the publisher.
