# Supplementary material for: Rapid biodegradation of atrazine by a novel Paenarthrobacter ureafaciens ZY and its effects on soil native microbial community dynamic
Source: Front Microbiol. 2023 Jan 4;13:1103168. doi: 10.3389/fmicb.2022.1103168 (PMC9846760; doi:10.3389/fmicb.2022.1103168)
Supplement: Supplementary file 1 [file Data_Sheet_1.docx]

**Supplementary Materials**

**Rapid biodegradation of atrazine by a novel *Paenarthrobacter ureafaciens* ZY and its effects on soil native microbial community dynamic**

Yue Zhao^1^, Xin Li^1^*, Yunyang Li^1^, Huanyu Bao^1^, Jun Nan^1^, Guoren Xu^1,2^

1. School of Environment, Harbin Institute of Technology, Harbin, 150090, China
2. College of Resources and Environment, University of Chinese Academy of Sciences (UCAS), Beijing, 100049, China

*Corresponding author: Xin Li

E-mail address: lixinwindows@163.com (Xin Li)

**Summary:**

**Figure S1.** The morphological characteristics of *P. ureafaciens* ZY observed on LB plate (A) and under microscope (B).

**Figure S2.** Scanning electron micrograph of strain *P. ureafaciens* ZY.

**Figure S3.** Carbon utilization pattern of *P. ureafaciens* ZY in the GEN III microplate of Biolog microstation system after 48 h of incubation.

**Figure S4.** The analytical data for the detected ions included (A) m/z 198.0, (B) m/z 171.0, and (C) m/z 128.0. Compounds (A), (B), and (C) were identifed as hydroxyatrazine, N-isopropylammelide, and cyanuric acid, respectively.

**Figure S5** Richness estimators and diversity indices of bacterial communities. Observed OTU numbers (A); Chao1 index (B); Simpson index (C); Shannon index (D);

**Figure S6** Agarose gel electrophoresis (1%) of PCR products of atrazine-degrading genes of strain ZY. (1: *trz*N; 2: *atz*B; 3: *atz*C; 4: *atz*A; 5: *atz*D; 6: *atz*E; 7: *atz*F; 8: *trz*D; M: DL 2000 DNA marker).

**Figure S7** Composition of co-occurrence networks based on the OTUs under atrazine alone treatments (A) and *P. ureafaciens* ZY inoculation treatments (B). The node denotes the selected OTUs.

**Table S1.** Sequence of the primer pairs used in this study.

**Table S2.** The phospholipid fatty acids classifications provided by the Sherlock Microbial Identification System.

**Table S3.** Carbon source metabolic characteristics of *P. ureafaciens* ZY in GEN III Microplate after 48 h of incubation.

**Table S4.** The different atrazine degradation abilities of various atrazine-degrading strains.

**Table S5.** Toxicity of atrazine and its metabolites as predicted by the ECOSAR based on QSAR

**Table S6.** Topological features in co-occurrence network of microbial community.


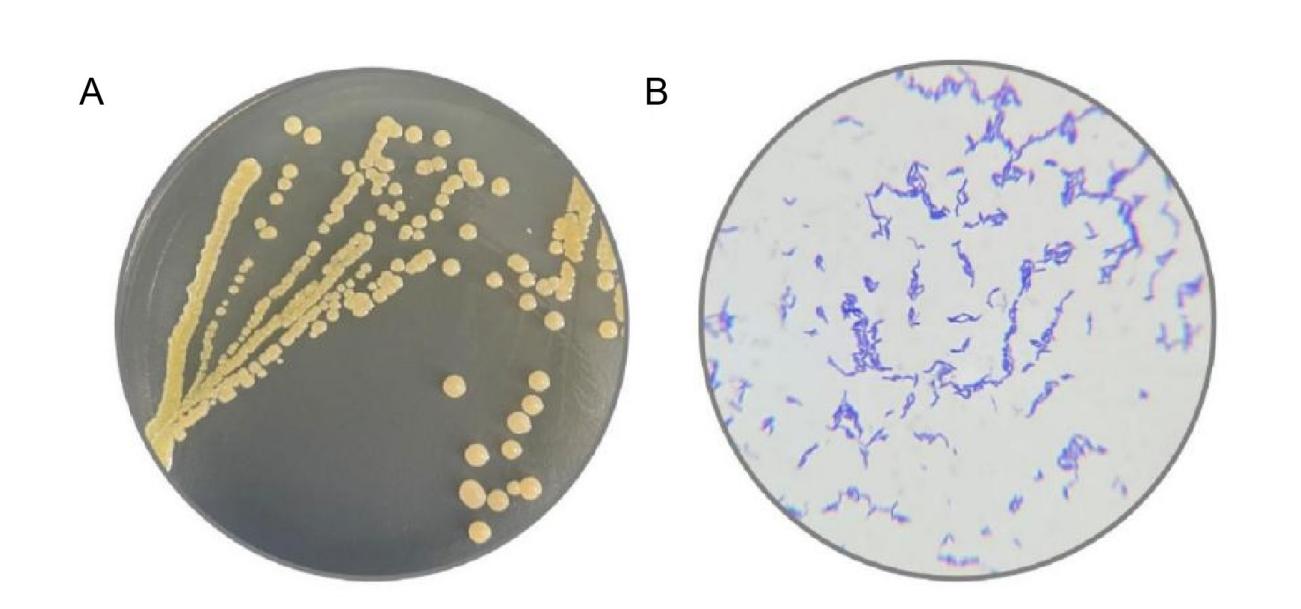


**Figure S1.** The morphological characteristics of *P. ureafaciens* ZY observed on LB plate (A) and under microscope (B).


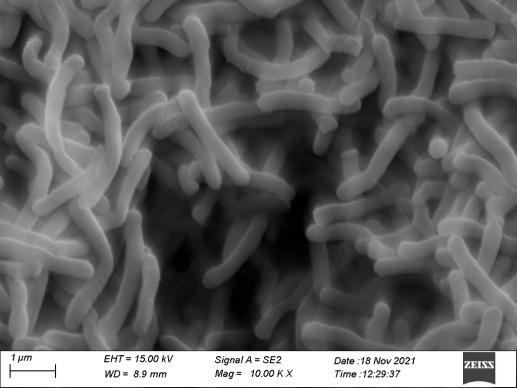


**Figure S2.** Scanning electron micrograph of strain *P. ureafaciens* ZY.


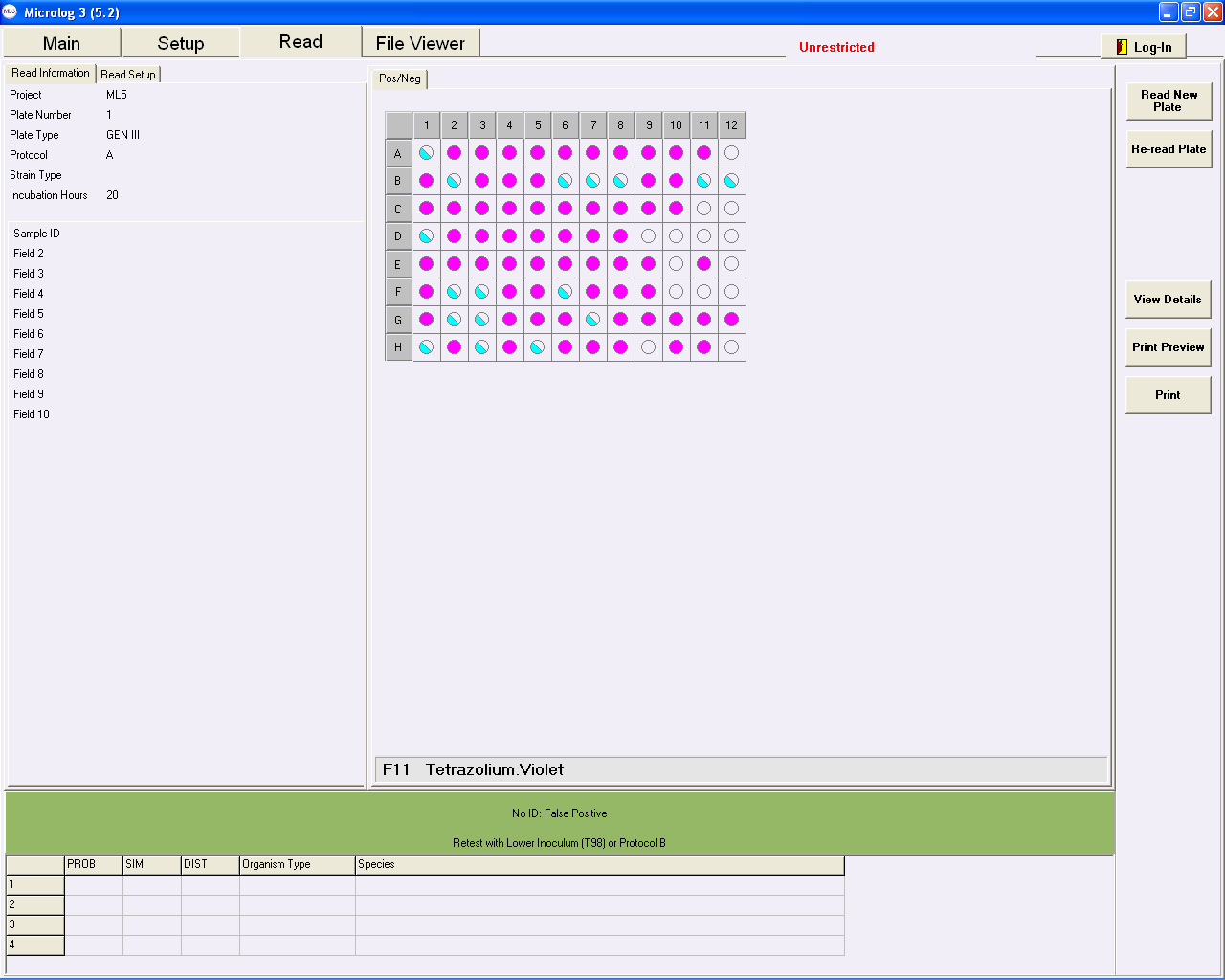


**Figure S3.** Carbon utilization pattern of *P. ureafaciens* ZY in the GEN III microplate of Biolog microstation system after 48 h of incubation.

**
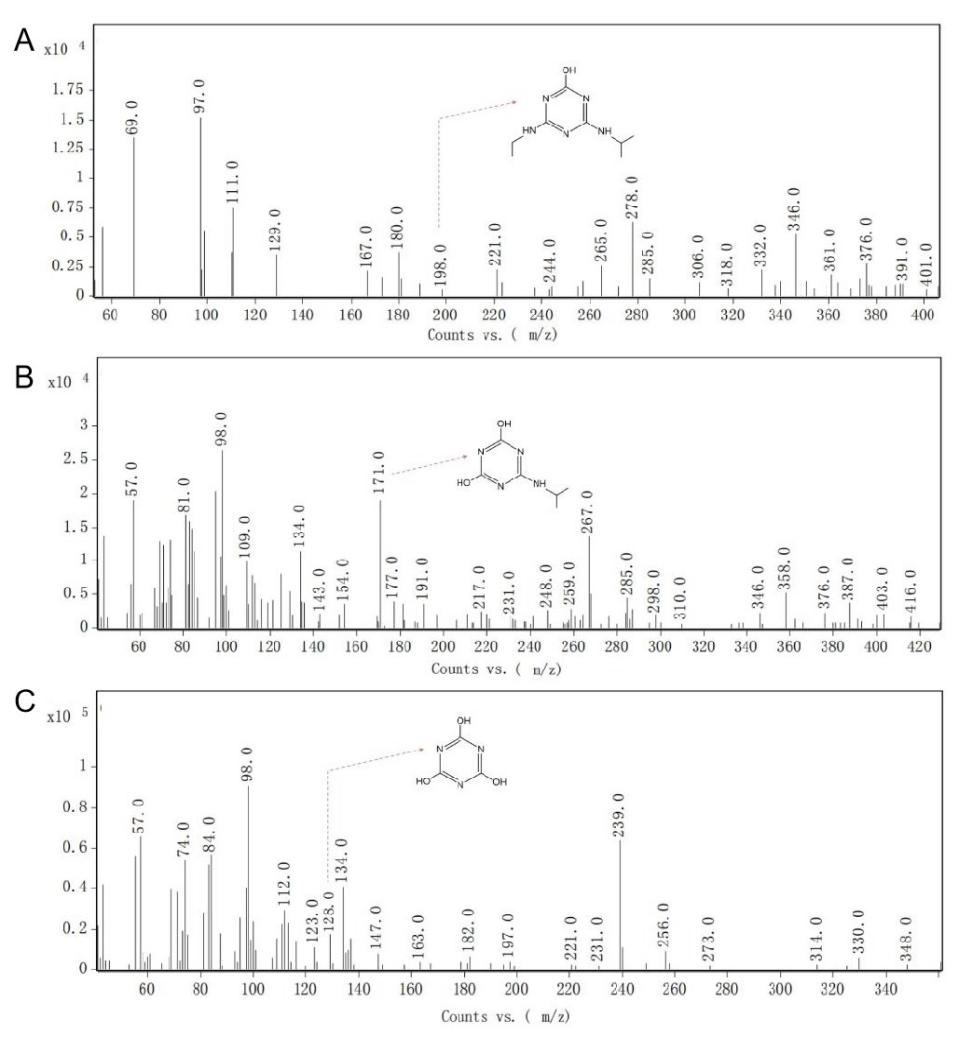
**

**Figure S4.** The analytical data for the detected ions included (A) m/z 198.0, (B) m/z 171.0, and (C) m/z 128.0. Compounds (A), (B), and (C) were identifed as hydroxyatrazine, N-isopropylammelide, and cyanuric acid, respectively.


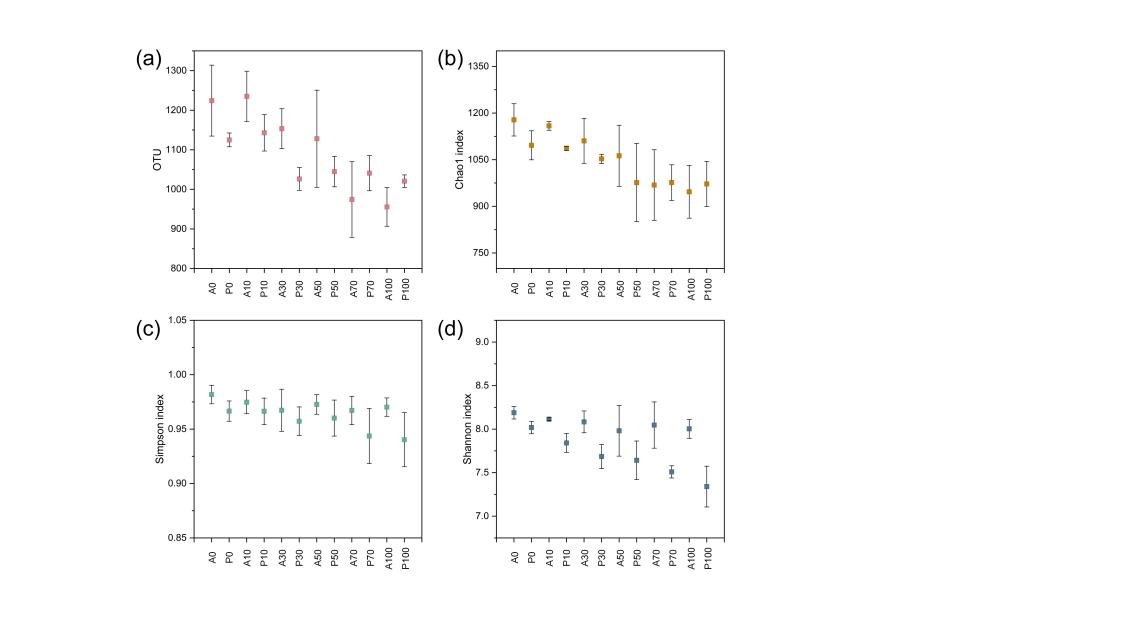


**Figure S5** Richness estimators and diversity indices of bacterial communities. Observed OTU numbers (A); Chao1 index (B); Simpson index (C); Shannon index (D); A0: Equivalent amount of methanol were added into native soil. A10: 10 mg kg^-1^ atrazine-contaminated soil; A30: 30 mg kg^-1^ atrazine-contaminated soil; A50: 50 mg kg^-1^ atrazine-contaminated soil; A70: 70 mg kg^-1^ atrazine-contaminated soil; A100: 100 mg kg^-1^ atrazine-contaminated soil; P0: Equivalent amount of methanol and *P. ureafaciens* ZY were added into native soil; P10: 10 mg kg^-1^ atrazine-contaminated soil with *P. ureafaciens* ZY inoculation; P30: 30 mg kg^-1^ atrazine-contaminated soil with *P. ureafaciens* ZY inoculation; P50: 50 mg kg^-1^ atrazine-contaminated soil with *P. ureafaciens* ZY inoculation; P70: 70 mg kg^-1^ atrazine-contaminated soil with *P. ureafaciens* ZY inoculation; P100: 100 mg kg^-1^ atrazine-contaminated soil with *P. ureafaciens* ZY inoculation;

**
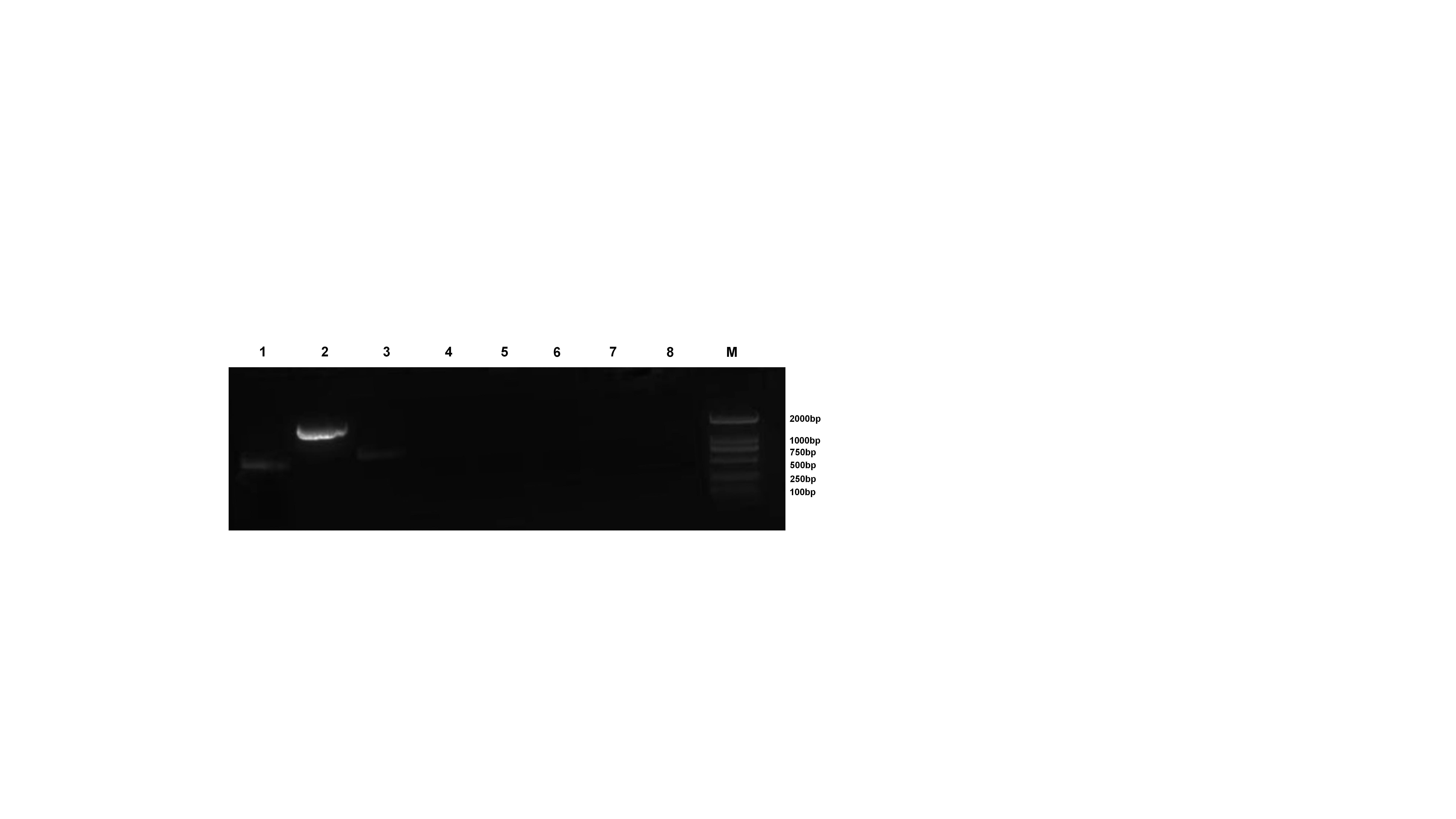
**

**Figure S6** Agarose gel electrophoresis (1%) of PCR products of atrazine-degrading genes of strain ZY. (1: *trz*N; 2: *atz*B; 3: *atz*C; 4: *atz*A; 5: *atz*D; 6: *atz*E; 7: *atz*F; 8: *trz*D; M: DL 2000 DNA marker).


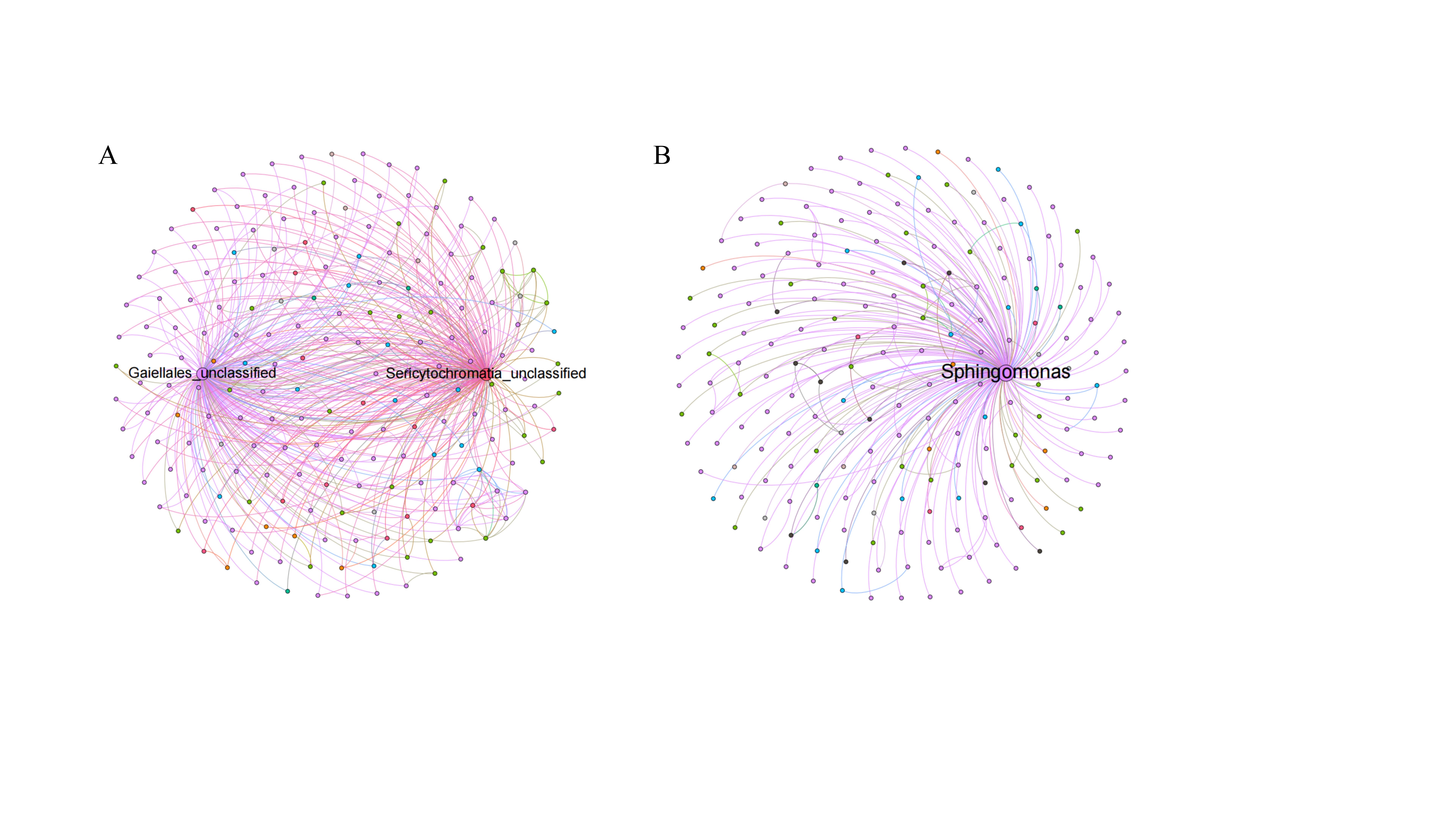


**Figure S7** Composition of co-occurrence networks based on the OTUs under atrazine alone treatments (A) and *P. ureafaciens* ZY inoculation treatments (B) treatments. The node denotes the selected OTUs.

**Table S1.** Sequence of the primer pairs used in this study (Sebaï et al., 2011).

| Gene | Primer | Sequence (5’→3’) | Annealing temperature (℃) |
| --- | --- | --- | --- |
| *atz*A | A1f | ACGGGCGTCAATTCTATGAC | 60 |
|  | A1r | CACCCACCTCACCATAGACC |  |
| *atz*B | B1f | AGGGTGTTGAGGTGGTGAAC | 60 |
|  | B1r | CACCACTGTGCTGTGGTAGA |  |
| *atz*C | Cf | GCTCACATGCAGGTACTCCA | 57 |
|  | C1r | TCCCCCAACTAAATCACAGC |  |
| *atz*D | Df | GGGTCTCGAGGATTTGATTG | 60 |
|  | Dr | TCCCACCTGACATCACAAAC |  |
| *atz*E | Ef | GAGCCTCTGTCCGTAGATCG | 60 |
|  | Er | GATGGCGTGTACCGTTTACC |  |
| *atz*F | Ff | ACCAGCCCTTGAATCATCAG | 60 |
|  | Fr | ACTTACAAACGCACCGAACC |  |
| *trz*N | C190-10 | CACCAGCACCTGTACGAAGG | 57 |
|  | C190-11 | GATTCGAACCATTCCAAACG |  |
| *trz*D | TrzDf1 | CCTCGCGTTCAAGGTCTACT | 60 |
|  | TrzDr1 | TCGAAGCGATAACTGCATTG |  |

**Table S2.** The phospholipid fatty acids classifications provided by the Sherlock Microbial Identification System.

| Category | Index | Multiplier | Peaks Microbial Types General Soil for PLFAD1 v1.31 |
| --- | --- | --- | --- |
| Non FAME | 0 | 1 | Phthalate 1, Phthalate 2 |
| General FAME | 0 | 1 | 10:0, 11:0, 12:0, 13:0, 15:0 aldehyde, 14:0, 16:1 w9c aldehyde, 16:0 aldehyde, 15:0, 16:1 w7c alcohol, 16:0 N alcohol, 16:0, 17:0, 18:0, 19:0, 20:0, 21:0, 22:0, 23:0, 24:0 |
| AM Fungi | 1 | 1 | 16:1 w5c |
| Gram Negative | 1 | 1 | 10:0 2OH, 10:0 3OH, 12:1 w8c, 12:1 w5c, 13:1 w5c, 13:1 w4c, 13:1 w3c, 12:0 2OH, 14:1 w9c, 14:1 w8c, 14:1 w7c, 14:1 w5c, 15:1 w9c, 15:1 w8c, 15:1 w7c, 15:1 w6c, 15:1 w5c, 14:0 2OH, 16:1 w9c, 16:1 w7c, 14:0 3OH, 16:1 w6c, 16:1 w4c , 16:1 w3c, 17:1 w9c, 17:1 w8c, 17:1 w7c, 17:1 w6c, 17:0 cyclo w7c, 17:1 w5c, 17:1 w4c, 17:1 w3c, 16:0 2OH, 18:0 cyclo w6c, 18:1 w8c, 18:1 w7c, 18:1 w6c, 18:1 w5c, 18:1 w3c, 19:1 w9c, 19:1 w8c, 19:1 w7c, 19:1 w6c, 19:0 cyclo w7c, 19:0 cyclo w6c, 20:1 w9c, 20:1 w8c, 20:1 w6c, 20:1 w4c, 20:0 cyclo w6c, 21:1 w9c, 21:1 w8c, 21:1 w6c, 21:1 w5c, 21:1 w4c, 21:1 w3c, 22:1 w9c, 22:1 w8c, 22:1 w6c, 22:1 w5c, 22:1 w3c, 22:0 cyclo w6c, 24:1 w9c, 24:1 w7c, 11:0 iso 3OH, 14:0 iso 3OH, 17:0 iso 3OH |
| Methanotroph | 1 | 1 | 16:1 w8c |
| Eukaryote | 1 | 1 | 15:4 w3c, 15:3 w3c, 16:4 w3c, 16:3 w6c, 18:3 w6c, 19:4 w6c, 19:3 w6c, 19:3 w3c, 20:4 w6c, 20:5 w3c, 20:3 w6c, 20:2 w6c, 21:3 w6c, 21:3 w3c, 22:5 w6c, 22:6 w3c, 22:4 w6c, 22:5 w3c, 22:2 w6c, 23:4 w6c, 23:3 w6c, 23:3 w3c, 23:1 w5c, 23:1 w4c, 24:4 w6c, 24:3 w6c, 24:3 w3c, 24:1 w3c, 18:4 w3c |
| Fungi | 1 | 1 | 18:2 w6c |
| Gram Positive | 1 | 1 | 11:0 iso, 11:0 anteiso, 12:0 iso, 12:0 anteiso, 13:0 iso, 13:0 anteiso, 14:1 iso w7c, 14:0 iso, 14:0 anteiso, 15:1 iso w9c, 15:1 iso w6c, 15:1 anteiso w9c, 15:0 iso, 15:0 anteiso, 16:0 iso, 16:0 anteiso, 17:1 iso w9c, 17:0 iso, 17:0 anteiso, 18:0 iso, 17:1 anteiso w9c, 17:1 iso w10c, 17:1 anteiso w7c, 18:1 w9c, 19:0 cyclo w9c, 19:0 iso, 19:0 anteiso, 20:0 iso, 22:0 iso |
| Anaerobe | 1 | 1 | 12:0 DMA, 13:0 DMA, 14:1 w7c DMA, 14:0 DMA, 15:0 iso DMA, 15:0 DMA, 16:2 DMA, 17:0 DMA, 16:1 w9c DMA, 16:1 w7c DMA, 16:1 w5c DMA, 16:0 DMA, 18:2 DMA, 18:1 w9c DMA, 18:1 w7c DMA, 18:1 w5c DMA, 18:0 DMA, 19:0 cyclo 9,10 DMA |
| Actinomycetes | 1 | 1 | 16:0 10-methyl, 17:1 w7c 10-methyl, 17:0 10-methyl, 22:0 10-methyl, 18:1 w7c 10-methyl, 18:0 10-methyl, 19:1 w7c 10-methyl, 20:0 10-methyl |

**Table S3.** Carbon source metabolic characteristics of *P. ureafaciens* ZY in GEN III Microplate after 48 h of incubation.

| Position | Carbon source | ZY | Position | Carbon source | ZY | Position | Carbon source | ZY | Position | Carbon source | ZY |
| --- | --- | --- | --- | --- | --- | --- | --- | --- | --- | --- | --- |
| A1 | Negativecontrol | W | B1 | D-Raffnose | + | C1 | a-D-Glucose | + | D1 | D-Sorbitol | W |
| A2 | Dextrin | + | B2 | D-Lactose | W | C2 | D-Mannose | + | D2 | D-Mannitol | + |
| A3 | Maltose | + | B3 | D-Melibiose | + | C3 | D-Fructose | + | D3 | L-Arabitol | + |
| A4 | D-trehalose | + | B4 | 3-MethylD-glucoside | + | C4 | D-Galactose | + | D4 | Myo-Inositol | + |
| A5 | D-cellobiose | + | B5 | D-Salicin | + | C5 | 3-Methylglucose | + | D5 | Glycerol | + |
| A6 | Gentobiose | + | B6 | N-AcetylD-glucosamine | W | C6 | D-Fucose | + | D6 | D-Glucose6phosphate | + |
| A7 | Sucrose | + | B7 | N-AcetylD-mannosamine | W | C7 | L-Fucose | + | D7 | D-fructose6-phosphate | + |
| A8 | D-Turanose | + | B8 | N-AcetylD-galactosamine | W | C8 | L-Rhamnose | + | D8 | Dasparticacid | + |
| A9 | Stachyose | + | B9 | N-AcetylD-neuraminicacid | + | C9 | Inosine | + | D9 | Dserine | - |
| A10 | Positivecontrol | + | B10 | 1% NaCl | + | C10 | 1% Sodiumlactate | + | D10 | Troleandomycin | - |
| A11 | pH-6.0 | + | B11 | 4%NaCl | W | C11 | Fusidicacid | - | D11 | Rifamycin | - |
| A12 | pH-0 | - | B12 | 8% NaCl | W | C12 | D-serine | - | D12 | Minocycline | - |
| E1 | Gelatin | + | F1 | pectin | + | G1 | Hydroxyphenylaceticacid | + | H1 | Tween40 | W |
| E2 | Glycl-L-proline | + | F2 | Dgalacturonicacid | W | G2 | Pyruvicacidmethylester | W | H2 | G-AmineNbutyricacid | + |
| E3 | L-alanine | + | F3 | Lgalactonicacidgalactone | W | G3 | D-LacticAcid | W | H3 | aHydroxylbutyricacid | W |
| E4 | L-Arginine | + | F4 | Dgluconicacid | + | G4 | L-Lacticacid | + | H4 | β-Hydroxylbutyricacid | + |
| E5 | L-Asparticacid | + | F5 | Dglucoronicacid | + | G5 | Citricacid | + | H5 | aketobutyricacid | W |
| E6 | L-Glutamicacid | + | F6 | glucuronamide | W | G6 | aKetoglutaricacid | + | H6 | Acetoaceticacid | + |
| E7 | L-Histidine | + | F7 | Mucicacid | + | G7 | D-Malicacid | W | H7 | Propionicacid | + |
| E8 | L-Pyroglumaticacid | + | F8 | Quinicacid | + | G8 | L-Malicacid | + | H8 | Aceticacid | + |
| E9 | L-Serine | + | F9 | Dsacchricacid | + | G9 | Bromosuccinicacid | + | H9 | Formicacid | - |
| E10 | Lincomycin | - | F10 | Vancomycin | - | G10 | Nalidixicacid | + | H10 | aztreonam | + |
| E11 | Guanidinehydroclanle | + | F11 | Tetrazoliumvoilet | - | G11 | Lithiumchloride | + | H11 | Sodiumbutyrate | + |
| E12 | Nicaproof4 | - | F12 | TetrazoliumBlue | - | G12 | Potassiumtellurite | + | H12 | Sodiumbromate | - |

“+” means, Positive, “W” means, weakly positive and “-” means, Negative.

**Table S4.** The different atrazine degradation abilities of various atrazine-degrading strains

| Strain | Initial concentration (mg L^-1^) | Time | Degradation rate | Degradation (%) | References |
| --- | --- | --- | --- | --- | --- |
| *Rhodococcus* sp. BCH2 | 100 | 7 d | 10.71 mg L^-1^ d^-1^ | 75 | Kolekar et al., 2014 |
| *Pseudomonas* sp. EGD-AKN5 | 100 | 3.6 d | 27.31 mg L^-1^ d^-1^ | 98.3 | Bhardwaj et al., 2015 |
| *Shewanella* sp. YJY4 | 100 | 1.5 d | 2.78 mg L^-1^ h^-1^ | 100 | Ye et al., 2016 |
| *Pseudomonas* sp. ADP | 100 | - | 4.17 mg L^-1^ h^-1^ | - | Mandelbaum et al., 1995 |
| *Arthrobacter* sp. DAT1 | 100 | - | 2.08 mg L^-1^ h^-1^ | - | Xie et al., 2013 |
| *Arthrobacter* sp.ZXY-2 | 100 | - | 9.52 mg L^-1^ h^-1^ | - | Zhao et al., 2018 |
| *Sinorihizobium* sp. K | 100 | 30 h | 3.33 mg L^-1^ h^-1^ | 100 | Chen et al., 2017 |
| *Ensifer* sp. CX-T | 100 | 30 h | 3.33 mg L^-1^ h^-1^ | 100 | Chen et al., 2017 |
| Co-culture of *Arthrobacter* sp. DNS10 and *Enterobacter* sp. P1 | 100 | 2 d | 2.07 mg L^-1^ h^-1^ | 99.18 | Jiang et al., 2019 |
| *Rhodococcus* sp. NI86/21 | 55 | 48 h | 1.15 mg L^-1^ h^-1^ | 100 | Nagy et al., 1995 |
| *Citricoccus* sp. TT3 | 50 | 2.75 d | 0.76 mg L^-1^ h^-1^ | 100 | Yang et al., 2018 |
| *Agrobacterium radiobacter* J14a | 50 | 48 h | 1.04 mg L^-1^ h^-1^ | 100 | Struthers, et al., 1998 |
| *Nocardioides* sp. EAA-3 | 25 | 60 h | 0.42 mg L^-1^ h^-1^ | 100 | Omotayo et al., 2013 |
| *Nocardioides* sp. EAA-4 | 25 | 72 h | 0.35 mg L^-1^ h^-1^ | 100 | Omotayo et al., 2013 |
| *Arthrobacter* sp. MCM B-436 | 25 | - | 0.83mg∙L^-1^∙h^-1^ | - | Vaishampayan et al., 2007 |
| *Arthrobacter* sp. C3 | 25 | - | 0.35 mg∙L^-1^∙h^-1^ | - | Wang et al., 2016 |
| *P. ureafaciens* ZY | 30 | 6 h | 4.98 mg∙L^-1^∙h^-1^ | 99.57 | In this study |
|  | 50 | 6 h | 6.86 mg∙L^-1^∙h^-1^ | 82.33 | In this study |
|  | 70 | 6 h | 8.67 mg∙L^-1^∙h^-1^ | 74.28 | In this study |
|  | 100 | 8 h | 12.5 mg∙L^-1^∙h^-1^ | 100 | In this study |

**Table S5.** Toxicity of atrazine and its metabolites as predicted by the ECOSAR based on QSAR

| Chemicals | Organism | Duration | End point | Concentration (mg L^-1^) |  |
| --- | --- | --- | --- | --- | --- |
| Atrazine | Fish | 96h | LC50 | 20.20658 |  |
|  | Daphnid | 48h | LC50 | 15.6713505 |  |
|  | Green Algae | 96h | EC50 | 0.10541339 |  |
|  | Fish |  | ChV | 1.5044174 |  |
|  | Daphnid |  | ChV | 0.91682744 |  |
|  | Green Algae |  | ChV | 0.51041096 |  |
|  | Fish (SW) | 96h | LC50 | 19.538902 |  |
|  | Mysid (SW) | 96h | LC50 | 4.6338477 |  |
|  | Fish (SW) |  | ChV | 1.5662321 |  |
|  | Mysid (SW) |  | ChV | 0.6867725 |  |
| Hydroxyatrazine | Fish | 96h | LC50 | 74.431335 | Chemical may not be soluble enough to measure this predicted effect. If the effect level exceeds the water solubility by 10X, typically no effects at saturation (NES) are reported. |
|  | Daphnid | 48h | LC50 | 39.95492 | Chemical may not be soluble enough to measure this predicted effect. If the effect level exceeds the water solubility by 10X, typically no effects at saturation (NES) are reported. |
|  | Green Algae | 96h | EC50 | 0.23944934 |  |
|  | Fish |  | ChV | 5.575917 |  |
|  | Daphnid |  | ChV | 2.666469 |  |
|  | Green Algae |  | ChV | 1.1006988 |  |
|  | Fish (SW) | 96h | LC50 | 57.30301 | Chemical may not be soluble enough to measure this predicted effect. If the effect level exceeds the water solubility by 10X, typically no effects at saturation (NES) are reported. |
|  | Mysid (SW) | 96h | LC50 | 16.828146 | Chemical may not be soluble enough to measure this predicted effect. If the effect level exceeds the water solubility by 10X, typically no effects at saturation (NES) are reported. |
|  | Fish (SW) |  | ChV | 3.3022342 |  |
|  | Mysid (SW) |  | ChV | 4.9547863 |  |
| N-Isopropylammelide | Fish | 96h | LC50 | 2629.0442 | Chemical may not be soluble enough to measure this predicted effect. If the effect level exceeds the water solubility by 10X, typically no effects at saturation (NES) are reported. |
|  | Daphnid | 48h | LC50 | 529.4874 |  |
|  | Green Algae | 96h | EC50 | 2.3328896 |  |
|  | Fish |  | ChV | 200.22221 |  |
|  | Daphnid |  | ChV | 50.185886 |  |
|  | Green Algae |  | ChV | 9.337214 |  |
|  | Fish (SW) | 96h | LC50 | 1102.7832 |  |
|  | Mysid (SW) | 96h | LC50 | 572.3261 |  |
|  | Fish (SW) |  | ChV | 26.378761 |  |
|  | Mysid (SW) |  | ChV | 1049.3342 |  |
| Cyanuric acid | Fish | 96h | LC50 | 63.642815 |  |
|  | Daphnid | 48h | LC50 | 31.83391 |  |
|  | Green Algae | 96h | EC50 | 0.1865983 |  |
|  | Fish |  | ChV | 4.7733707 |  |
|  | Daphnid |  | ChV | 2.1788788 |  |
|  | Green Algae |  | ChV | 0.84924006 |  |
|  | Fish (SW) | 96h | LC50 | 46.899742 |  |
|  | Mysid (SW) | 96h | LC50 | 14.349796 |  |
|  | Fish (SW) |  | ChV | 2.5368178 |  |
|  | Mysid (SW) |  | ChV | 4.820129 |  |

**Table S6**. Topological features in co-occurrence network of microbial community.

| Network indexes | Atrazine alone treatments | *P. ureafaciens* ZY inoculation treatments |
| --- | --- | --- |
| Nodes | 201 | 201 |
| edges | 500 | 246 |
| ACC | 0.99 | 0.984 |
| APL | 1.975 | 1.988 |
| Diameter | 2 | 2 |
| Modularity | 0.258 | 0.27 |
| Density | 0.025 | 0.012 |

APL, average path length; ACC, average clustering coefficient.

**Reference**

Bhardwaj, P., Sharma, A., Sagarkar, S., and Kapley, A. (2015). Mapping atrazine and phenol degradation genes in *Pseudomonas* sp. EGD-AKN5. *Biochem. Eng. J.* 102, 125-134. doi: 10.1016/j.bej.2015.02.029

Chen, S. S., Yang, P. P., Kumar, J. R., Liu, Y., and Ma, L. M. (2017). Inconsistent carbon and nitrogen isotope fractionation in the biotransformation of atrazine by *Ensifer* sp. CX-T and *Sinorihizobium* sp. K. *Int. Biodeterior. Biodegradation* 125, 170-176. doi: 10.1016/j.ibiod.2017.09.014

Jiang, Z., Zhang, X. Y., Wang, Z. Y., Cao, B., Deng, S. J., Bi, M. C., et al. (2019). Enhanced biodegradation of atrazine by *Arthrobacter* sp . DNS10 during co-culture with a phosphorus solubilizing bacteria: *Enterobacter* sp. P1. *Ecotoxicol. Environ. Saf.* 172, 159-166. doi: 10.1016/j.ecoenv.2019.01.070

Kolekar, P. D., Phugare, S. S., and Jadhav, J.P. (2014). Biodegradation of atrazine by *Rhodococcus* sp. BCH2 to N-isopropylammelide with subsequent assessment of toxicity of biodegraded metabolites. *Environ. Sci. Pollut. Res.* 21, 2334-2345. doi: 10.1007/s11356-013-2151-6

Mandelbaum, R. T., Allan, D. L., and Wackett, L. P. (1995). Isolation and characterization of a *Pseudomonas* sp. that mineralizes the *s*-triazine herbicide atrazine. *Appl Environ Microbiol.* 61, 1451-1457. doi: 10.1128/aem.61.4.1451-1457.1995

Nagy, I., Compernolle, F., Ghys, K. J., Vanderleyden, J., and Mot, D. R. (1995). A Single cytochrome P-450 system is involved in degradation of the herbicide EPTC (*s*-ethyl dipropylthiocarbamate) and atrazine by *Rhodococcus* sp. Strain NI86/21. *Appl. Environ. Microbiol.* 61, 2056-2060. doi: 1128/aem.61.5.2056-2060.1995

Omotayo, A. E., Ilori, M. O., Radosevich, M., and Amund, O. O. (2013). Metabolism of atrazine in liquid cultures and soil microcosms by *Nocardioides* strains isolated from a contaminated Nigerian agricultural soil. *Soil Sediment Contam* 22, 365-375. doi: 10.1080/15320383.2013.733444

Sebaï, T. E., Devers-Lamrani, M., Changey, F., Rouard, N., and Martin-Laurent, F. (2011). Evidence of atrazine mineralization in a soil from the Nile Delta: isolation of *Arthrobacter* sp. TES6, An atrazine-degrading strain. *Int. Biodeterior. Biodegradation* 65, 1249-1255. doi: 10.1016/j.ibiod.2011.05.011

Struthers, J. K., Jayachandran, K., and Moorman, T. B. (1998). Biodegradation of atrazine by *Agrobacterium radiobacter* J14a and use of this strain in bioremediation of contaminated soil. *Appl. Environ. Microbiol.* 64, 3368-3375. doi: 10.1128/AEM.64.9.3368-3375.1998

Vaishampayan, P. A., Kanekar, P. P., and Dhakephalkar, P. K. (2007). Isolation and characterization of *Arthrobacter* sp. strain MCM B-436, an atrazine-degrading bacterium, from rhizospheric soil. *Int. Biodeterior. Biodegradation* 60, 273-278. doi: 10.1016/j.ibiod.2007.05.001

Wang, H., Liu, Y., Li, J., Lin, M., and Hu, X. (2016). Biodegradation of atrazine by *Arthrobacter* sp C3, isolated from the herbicide-contaminated corn field. *Int J Environ Sci Te.* 13, 257-262. doi: 10.1007/s13762-015-0860-8

Xie, S. G., Wan, R., Wang, Z., and Wang, Q. F. (2013). Atrazine biodegradation by *Arthrobacter* strain DAT1: efect of glucose supplementation and change of the soil microbial community. *Environ. Sci. Pollut. Res.* 20, 4078-4084. doi: 10.1007/s11356-012-1356-4

Yang, X., Wei, H., Zhu, C., and Geng, B. (2018). Biodegradation of atrazine by the novel *Citricoccus* sp. strain TT3. *Ecotoxicol. Environ. Saf.* 147, 144-150. doi: 10.1016/j.ecoenv.2017.08.046

Ye, J. Y., Zhang, J. B., Gao, J. G., Li, H. T., Liang, D., and Liu, R. M. (2016). Isolation and characterization of atrazine-degrading strain *Shewanella* sp . YJY4 from cornfield soil. *Lett. Appl. Microbiol.* 6, 45-52. doi: 10.1111/lam.12584

Zhao, X. Y., Wang, L., Ma, F., and Yang, J. X. (2018). Characterisation of an efficient atrazine-degrading bacterium, *Arthrobacter* sp. ZXY-2: an attempt to lay the foundation for potential bioaugmentation applications. *Biotechnol. Biofuels* 11, 113. doi: 10.1186/s13068-018-1113-0
